# Nearly T2T, phased genome assemblies of corals reveal haplotype diversity and the evolutionary process of gene expansion

**DOI:** 10.1093/dnares/dsaf017

**Published:** 2025-07-01

**Authors:** Takeshi Takeuchi, Yoshihiko Suzuki, Eiichi Shoguchi, Manabu Fujie, Mayumi Kawamitsu, Chuya Shinzato, Noriyuki Satoh, Eugene W Myers

**Affiliations:** Marine Genomics Unit, Okinawa Institute of Science and Technology Graduate University, Onna, Okinawa 904-0495, Japan; Algorithms for Eco and Evo Genomics Unit, Okinawa Institute of Science and Technology Graduate University, Onna, Okinawa 904-0495, Japan; Marine Genomics Unit, Okinawa Institute of Science and Technology Graduate University, Onna, Okinawa 904-0495, Japan; Sequencing Section, Okinawa Institute of Science and Technology Graduate University, Onna, Okinawa 904-0495, Japan; Sequencing Section, Okinawa Institute of Science and Technology Graduate University, Onna, Okinawa 904-0495, Japan; Atmosphere and Ocean Research Institute, The University of Tokyo, Kashiwa 277-8564, Japan; Marine Genomics Unit, Okinawa Institute of Science and Technology Graduate University, Onna, Okinawa 904-0495, Japan; Algorithms for Eco and Evo Genomics Unit, Okinawa Institute of Science and Technology Graduate University, Onna, Okinawa 904-0495, Japan

**Keywords:** genome, haplotype-phased genome assembly, coral, gene duplication, immunity

## Abstract

Gene family expansion illustrates a critical aspect of evolutionary adaptation. However, the mechanisms by which gene family expansions emerge and are maintained in the genome remain unclear. Here, we report de novo, nearly telomere-to-telomere (T2T), haplotype-phased genome assemblies of 2 coral species, *Acropora tenuis* and *Acropora digitifera*. By comparing haplotypes within a single individual and across species, we identified genomic regions spanning several megabases with highly disordered gene arrangements, termed non-syntenic regions (nSRs). In these nSRs, there are clusters of genes that emerged by lineage-specific gene family expansion. The gene repertoire within nSRs exhibits significant sequence diversity and distinct expression patterns, suggesting functional diversification. We propose that lineage-specific gene family expansion in nSRs occurs through recurrent tandem duplications mediated by non-allelic homologous recombination (NAHR) events, with nSRs serving as reservoirs for a diverse gene repertoire advantageous for survival. The nearly T2T-phased genomes provide new insights into the remarkable flexibility of genome organization and the evolution of gene family expansions.

## Introduction

Gene duplications and their subsequent divergence are a principal mechanism for generating genes with novel functions.^1^ Gene family expansion refers to a phenomenon where a specific gene family is duplicated multiple times, leading to an increase in the copy number of genes in a genome. It allows for the establishment of new genes, which can mutate to have different functions. This enables organisms to acquire functional and genetic diversity across the gene family, potentially contributing to the enhancement of an organism’s ability to adapt to environmental changes.

Lineage-specific gene duplication is a fundamental mechanism that drives species diversification and the evolution of unique traits, playing a pivotal role in adaptation. However, it remains unclear in the evolutionary process how the gene copy number is increased, and how the expanded gene family is maintained in the genome.

Whole genome sequencing has revealed that lineage-specific gene expansions occurred and are occurring in a variety of organisms. One prominent example is the expansion of the NLR [nucleotide-binding domain leucine-rich repeat (LRR) containing, also known as NOD-like receptors] gene family, which has been observed in various animals, such as fish,^2^ the amphioxus *Branchiostoma floridae*,^3^ the sponge *Amphimedon queenslandica*,^4^ the sea urchin *Strongylocentrotus purpuratus*,^5^ the pearl oyster *Pinctada fucata*,^6^ and the stony coral *Acropora digitifera*.^7^ Given their distant phylogenetic relationships, the gene family expansions clearly occurred independently in each lineage. Since the domain architectures of the NLR family across different organisms are comparable, they are thought to have similar biological functions, such as sensing molecules to detect intracellular infections or stress and activating innate immunity. Lineage-specific gene family expansion suggests adaptive genome evolution in response to specific environmental pressures, such as exposure to distinct pathogens.

Recently, we successfully constructed the haplotype-phased genome assembly of the pearl oyster *P. fucata*.^6^ This high-quality phased assembly revealed the existence of non-syntenic regions (nSRs), spanning several megabases, where gene arrangements are not conserved between haplotypes. These nSRs harbour a significant gene repertoire of NLRs. Similar gene family expansions of NLRs in genomic regions with large-scale structural variations (SVs) have also been reported in zebrafish.^8^ More than 1,500 NLRs have been identified across multiple wild and laboratory zebrafish populations, constituting a substantial NLR repertoire within the species.^9^ These findings raise a question of whether the large-scale gene expansions observed in nSRs are a phenomenon restricted to bivalves and fish or if they are widespread across other organisms.

An exemplary case of lineage-specific expansion across various gene families is observed in scleractinian corals, making them an ideal research model for studying gene family expansion. Since the genome of *A. digitifera* was first sequenced,^10^ genomic data sets from more than 30 scleractinian coral species have been accumulated.^11–24^ Comparative analyses, both within corals and among scleractinians and other cnidarians, have revealed that lineage-specific gene family expansions characterize coral genomes.^10,12,13,21,23,25^ Notable expansions are evident in several gene families, including NLRs,^7,26^ Toll-like receptors (TLRs),^10^ small heat shock proteins (HSP20),^13^ and fluorescent proteins (FPs).^27^ NLRs and TLRs are crucial components of innate immunity, detecting a wide array of endogenous molecules and triggering downstream signalling pathways. FPs are believed to fulfil multiple roles, such as photoprotection and oxidative stress reduction.^28–30^ Overall, the expanded gene families are often associated with innate immunity and resilience to environmental fluctuations. The complexity of innate immunity genes may be responsible for not only defence against pathogens but also their symbiosis with dinoflagellates and colonial body plan, which necessitate mechanisms for immune specificity and allorecognition. Coral gene expansions are also likely attributable to their sessile lifestyle, which requires resilience to fluctuating shallow marine environments.

In the present study, we report de novo haplotype-phased genome assemblies of 2 coral species, *Acropora tenuis* and *A. digitifera*. Due to the nearly complete, telomere-to-telomere (T2T) quality of the assembly, we successfully identified novel nSRs and SVs between haplotypes with high precision. Species-specific gene family expansions and copy number variations driven by extensive tandem duplications are evident in these nSRs. We observe significant expansions of gene families in nSRs that are likely involved in innate immunity and resistance to environmental stressors. The phased genome assemblies reveal remarkable genomic flexibility and diversity between haplotypes, providing valuable insights into genome evolution.

## Materials and methods

### Materials

For de novo genome sequencing of *A. tenuis* and *A. digitifera*, individuals kept in an aquaculture in Onna village were used to obtain sperm under a permit from the Okinawa Prefectural Government (No. 27-12). The samples were immediately frozen in liquid nitrogen and preserved at −80 °C until DNA extraction. High-molecular-weight genomic DNA was extracted using a NucleoBond HMW DNA Kit (MACHEREY-NAGEL, Germany) following the manufacturer’s instructions. The size distribution and concentration of the extracted DNA were assessed using a FEMTO Pulse (Agilent Technologies, CA, USA) and a Qubit Fluorometer (Thermo Fisher Scientific, MA, USA) devices. For HiFi and Omni-C sequencing, sperm DNA from the same individual was used. We used sperm for the genome sequencing as it is free from contamination of symbiont DNA. Although each sperm cell has different haplotypes as a result of recombination, the difference among cells is averaged out as negligible noise in the assembly process. Therefore, it is unlikely to affect constructing haplotype-phased genome assembly.

### Genome sequencing and assembly

The genomic DNA underwent fragmentation to achieve a target size of approximately 22 kb using a Megaruptor3 (Diagenode, Belgium). Fragmented DNA was subjected to a purification process using AMPure PB (Pacific Biosciences, CA, USA). The sizes of the resulting DNA fragments were verified using a Femto Pulse System (Agilent Technologies). The construction of HiFi SMRTbell libraries was accomplished using a SMRTbell Express Template Prep Kit 2.0 following the manufacturer’s protocol. Single-molecule sequencing was performed in CCS mode on a PacBio Sequel II platform. Post-sequencing, the integrity and quality of the obtained HiFi reads were ascertained through a k-mer spectra analysis (where k = 40), employing GeneScope.FK. (https://github.com/thegenemyers/GENESCOPE.FK) and PloidyPlot (https://github.com/thegenemyers/MERQURY.FK).

Based on the k-mer spectra analysis, a small fraction of the input DNA sequences of the *A. digitifera* sample was likely derived from another individual. Therefore, HiFi reads are filtered to obtain DNA sequences only from a single individual using purge tips (https://github.com/yoshihikosuzuki/purge_tips) with the default parameters, which iteratively remove reads mapped to contigs with a low sequencing coverage.

In order to develop a chromosome-scale assembly, long-range linkage information was procured based on an in vivo chromatin conformation capture library produced with Dovetail’s endonuclease-based, proximity-ligation method called Omni-C. The library underwent sequencing on an Illumina HiSeqX platform. The quality of the Omni-C reads was verified k-mer spectral methods GeneScope.FK and PloidyPlot with k = 21.

Both HiFi and Omni-C reads were then assembled together using hifiasm version 0.16^31^ in the Hi-C mode with the default parameter settings. The primary contigs for each of the 2 haplotypes produced by hifiasm were further scaffolded with Omni-C data as follows. First, the Omni-C reads were aligned to the primary contigs of both haplotypes using bwa (https://github.com/lh3/bwa) and divided into a separate read set for each haplotype based on whether they were mapped to contig(s) of that haplotype, while allowing assignments of a read to both haplotypes. Then, for each haplotype, scaffolding process was subsequently undertaken using SALSA (ver. 2.3).^32^ Following the establishment of the SALSA scaffolds, any remaining observable structural errors were eliminated through manual examination of the Omni-C contact map using Juicebox Assembly Tools,^33^ ensuring the structural integrity and accuracy of the final assembly.

To evaluate the quality of the final assembly, we basically followed the criteria proposed in the Vertebrate Genome Project.^34^ We ran Merqury (v1.3)^35^ on the HiFi reads to calculate the k-mer (k = 20)-based quality value (QV). To estimate structural accuracy, we calculated the reliable block N50 length using Asset (v1.0.3; https://github.com/dfguan/asset). For the Asset pipeline, HiFi reads and Omni-C reads were mapped with Winnowmap (V2.0.3)^36^ and bwa (v0.7.17),^37^ respectively, using default parameters.

### Repeat analysis

To develop a de novo repeat library, we utilized RepeatModeler (version 2.0.1),^38^ which incorporates ab initio repeat prediction programs (RepeatScout 1.0.6^39^ and RECON 1.08^40^). In addition, identification of Long Terminal Repeat (LTR) elements was carried out using LTRharvest^41^ and LTR_retriever.^42^ These repeats were classified based on BLAST hits to the Repbase library (ver. 20181026) (https://www.girinst.org/) using RepeatMasker ver. 4.1.0 (http://www.repeatmasker.org/). Transposable elements (TEs) not classified by RepeatMasker were further analysed using DeepTE.^43^ The Kimura substitution rates of TEs were computed using the Perl script calcDivergenceFromAlign.pl, which is included in RepeatMasker. Tandem repeats were detected using Tandem Repeat Finder (ver. 4.09)^44^ and subsequently classified with the Tandem Repeats Analysis Program (ver. 1.1.0).^45^

### RNA-seq-guided and de novo gene predictions

For Iso-Seq sequencing, total RNA was extracted from 4 frozen adult specimens employing TRIzol reagent (Life Technologies, Carlsbad, CA, USA), following the protocol specified by the manufacturer. The quality and quantity of the isolated RNA were assessed using a Bioanalyzer (Agilent Technologies) and quantified using a Qubit fluorometer (Life Technologies, Waltham, MA, USA). Double-stranded cDNA was constructed using a NEBNext Single Cell/Low Input cDNA Synthesis and Amplification Module (New England Biolabs, USA), according to PacBio instructions. Size selection of the PCR product was performed using ProNex Size-Selective Chemistry (Promega Corporation, USA), and fragments ranging from 2 to 8 kb were retained. Each SMRTbell library was constructed using the Pacific Biosciences SMRTbell Express Template Prep Kit 2.0. Fragment size distribution was verified using a Bioanalyzer High Sensitivity DNA Chip (Agilent Technologies) and quantified using a Qubit Fluorometer (Thermo Fisher Scientific). Iso-Seq sequencing was conducted using a PacBio Sequel II instrument, equipped with a Sequel II Sequencing 2.0 Kit and a SMRT Cell 8M Tray. Iso-Seq data processing was performed using the IsoSeq 3.1 software pipeline (Pacific Biosciences). This involved the generation of Circular Consensus Sequences (CCSs) from subreads. Full-length non-concatemer reads were selected and clustered to obtain full-length isoforms. These polished isoforms were then employed in subsequent downstream analyses.

For *A. tenuis* gene prediction, Iso-Seq reads along with RNA-seq short reads^25^ were integrated into the PASA (ver. 2.5)^46^ pipeline for training and testing for gene prediction using Augustus (ver. 3.3.3).^47^ For *A. digitifera*, Iso-Seq reads and RNA-seq short reads^10^ were fed into PASA. In addition, gene prediction information of *A. tenuis* was employed as a hint file for *A. digitifera* gene prediction using GeMoMa (ver. 1.9).^48^ The genome assemblies, after masking repeat sequences, were used for the gene prediction.

Gene models were then analysed using the InterProScan (ver. 5.14-53.0) platform^49^ to identify functional domains. We also ran BUSCO (ver. 4.1.2) with odb_metazoa10 database^50^ to assess the completeness of the gene models. To identify single-copy orthologs (SCOs) among the given taxa, orthoMCL (ver. 2.0.9)^51^ was used with its default parameters. SCOs of cnidarian animals (*A. tenuis*, *A. digitifera*, *Nematostella vectensis*, and *Hydra magnipapillata*) were identified using orthoMCL.

### Syntenic analysis

Syntenic blocks between 2 haplotypes were searched using MCScanX^52^ which applies Blastp results and gene arrangements with the following parameters: match_score: 50, match_size: 10, gap_penalty: − 1, overlap_window: 5, E_value: 1 × 10^−5^, max gaps: 25. *K*_a_ and *K*_s_ values of syntenic alleles between haplotypes or genomes were calculated using the add_kaks_to_MCScanX.pl script provided in the collinearity package (https://github.com/reubwn/collinearity). Using MCScanX, we determined genes that maintained gene order conservation across haplotypes. Genes in both haplotypes with a *K*_a_*K*_s_ value below 0.3 were considered homologous genes. Then, nSRs were identified based on a genome-wide window search with a 500 kb window size and 100 kb step size. A window containing more than 14 genes, with less than 15% of them being homologous was deemed non-syntenic. Over-represented Pfam domains in nSRs were identified using a hypergeometric test, adjusted using the Benjamini–Hochberg method in R.

### Molecular phylogeny

For the construction of a molecular phylogeny of functional domains, domain sequences were searched using InterProScan. For TEs, TE sequences in the genome assemblies are identified using RepeatMasker and DeepTE. Multiple sequence alignments of amino acid sequences or nucleotide sequences were made using MAFFT (ver. 7.271)^53^ with default parameters. Phylogenetic analyses were conducted using the maximum likelihood (ML) method in RAxML-NG (ver. 1.0.2).^54^ Reliability of the topology was checked by bootstrap analysis on the basis of 100 replicates.

### Data visualization

The pairwise alignments of chromosomal scaffolds were generated using d-genies.^55^ The synteny map, distribution of genes and nSRs, dot plots, and gene expression heatmaps were visualized using R custom scripts with ggplot2 library.^56^ Phylogenetic trees were drawn using R library ggtree.^57^

## Results

### Genome assembly

**Fig. 1.**
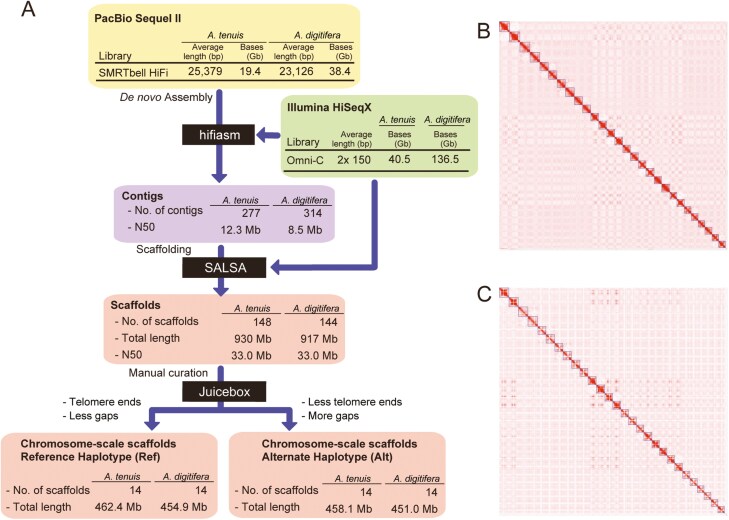
Haplotype-phased genome assemblies of *Acropora tenuis* and *Acropora digitifera*. (A) The sequencing and assembly pipeline to produce the haplotype-phased genome assembly. (B and C) A whole genome contact map of *A. tenuis* (B) and *A. digitifera* (C), showing 28 clusters representing chromosomal scaffolds. The colour scale is based on the relative interaction value from the highest (1, red) to the lowest value (<0.001, white).

We obtained 19.4 giga base pairs (Gbp) of HiFi long reads for *A. tenuis* and 38.4 Gbp for *A. digitifera* with estimated coverage depths of 42.2× and 83.5×, respectively (Fig. 1A, Table S1). The initial assemblies generated using the hifiasm software, comprised 277 contigs for *A. tenuis* and 314 contigs for *A. digitifera*, with the N50 length being 12.3 and 8.5 megabases (Mb). The contigs were further scaffolded using Omni-C read data, yielding 148 scaffolds for *A. tenuis* and 144 scaffolds for *A. digitifera* (Table S2).

The N50 values of both assemblies reached approximately 33 Mbp, and 28 of the scaffolds exhibited lengths greater than 26 Mbp (Fig. 1, Tables S3 and S4). Since the haploid genomes of *A. tenuis* and *A. digitifera* each have 14 chromosomes, these 28 scaffolds are thought to correspond to the chromosomes of the diploid genomes. The overall scaffold lengths are 929,860,843 bp in *A. tenuis* and 916,955,618 bp in *A. digitifera*, while the total lengths of the 28 chromosome-scale scaffolds are 920,427,833 bp and 905,948,004 bp, respectively. This means almost 99% of the sequences are contained within these chromosome-scale scaffolds in both genome assemblies. Analysis with Merqury using HiFi reads ascertained the k-mer-based QV of the assemblies to be 68.6, and k-mer completeness reached 99% (Table S2). Moreover, the N50 length of reliable haplotype-phased blocks, which are presumed to be devoid of structural errors, was estimated by the Asset pipeline to be 11.5 Mbp for *A. tenuis* and 8.0 Mbp for *A. digitifera*.

It is notable that the input genomic DNA was collected from sperm cells, which contain haplotype switching events due to recombination at a very low frequency. Recombination occurs randomly across the genome. Therefore, when focussing on a specific genomic region, only a small fraction of sperm is expected to carry DNA in which recombination has occurred within that region. Consequently, sequence reads containing recombination were treated as low-coverage chimeric reads and were appropriately excluded during the assembly process. If recombination hotspots are present in the coral genome and the same recombination event occurs at a hotspot in multiple reads, reads with recombination may not be excluded. Even in such cases, however, in the assembly graph the coverage of paths formed by reads with recombination should remain low. Since only high-coverage major paths are incorporated into the final assembly, the impact of recombination is likely eliminated. Indeed, both the QV and the N50 length of reliable haplotype-phased blocks indicate that the assemblies are highly accurate and that the small fraction of SVs among sperm DNA had only a little influence on the assembly process.

For the 2 chromosome-scale genome assemblies, the *A. tenuis* assembly exhibits 1 to 12 gaps per chromosomal scaffold, and the *A. digitifera* assembly presents 1 to 11 gaps per chromosomal scaffold (Tables S3 and S4). The chromosomal scaffolds often contain telomere repeat motif (TTAGGG), which is conserved among metazoans^58^ (Figs. S1–S4). Among the 28 chromosomal scaffolds of 2 coral diploid genome assemblies, telomere sequences were identified in 24 scaffolds for *A. tenuis* and 25 scaffolds for *A digitifera*. The longest telomere sequence reached 20 kb on chromosome 1 of *A. tenuis* and 16 kb at chromosome 9 of *A. digitifera*. These metrics collectively underscore the superior quality of the diploid assembly. Therefore, these assemblies demonstrate a level of completeness that far surpasses that of previous versions of *Acropora* genomes.^10,20,21^

Pairwise alignment of the scaffolds performed using d-genies resulted in the identification of 14 paired chromosomal scaffolds (Figs. S5 and S6). We assigned the 2 scaffolds corresponding to a pair of homologous chromosomes as either reference (Ref) or alternate (Alt) haplotype, based on the following criteria (Tables S3 and S4). (i) If only one of the haplotypes has telomere sequences at both ends of a scaffold, that haplotype is assigned as a reference haplotype. (ii) If both haplotypes have telomere sequences at both ends, the one with fewer gaps is assigned as the reference haplotype. (iii) If both haplotypes have telomere sequences on only one side or none on both sides, the haplotype with fewer gaps is assigned as a reference haplotype. The total lengths of the Ref and Alt haploid genome assemblies are 462.4 and 458.1 Mb for *A. tenuis* and 454.9 and 451.0 Mb for *A. digitifera*, respectively (Fig. 1A, Table S2). These values are comparable to the genome size of *A. digitifera* (420 Mb) estimated by flow cytometry.^10^

The integrity of the haplotype-phased assemblies of both species was assessed using Benchmarking Universal Single-Copy Orthologs (BUSCO) analysis with the metazoan dataset (Table S2). Both of the 2 haploid genome assembly of *A. tenuis* and *A. digitifera* contained more than 94.4% of complete and single-copy BUSCOs. Thus, these genome assemblies provide a reliable basis for precise comparisons of gene counts both between haplotypes and between species. Taking these results together, we conclude that we have successfully constructed haplotype-phased, nearly complete T2T genome assemblies of the 2 coral species.

### Identification of nSRs

**Fig. 2.**
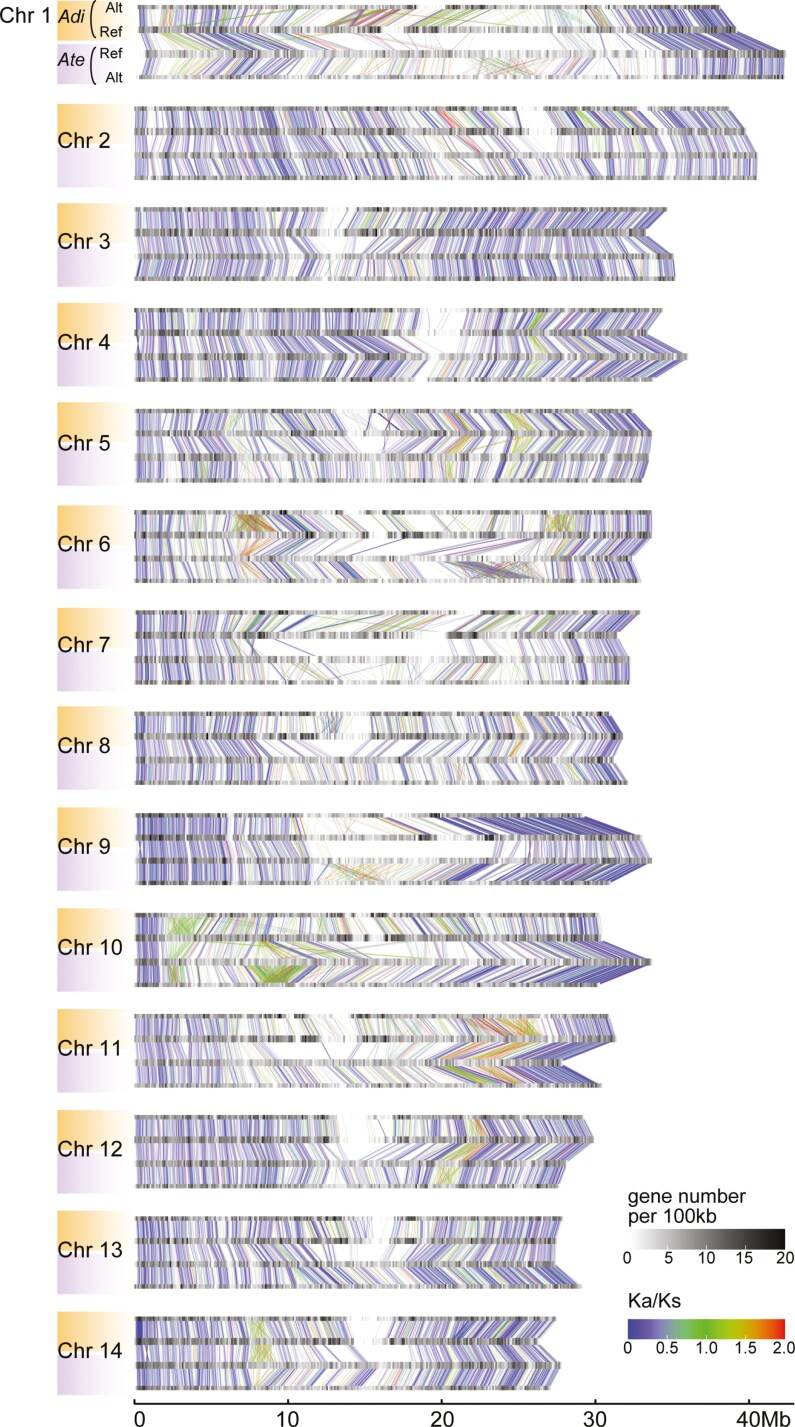
Collinearity between the 14 chromosomal scaffolds of reference (Ref) and alternate (Alt) haplotypes of *Acropora tenuis* (Ate) and *Acropora digitifera* (Adi). The chromosomes are represented by grey to black gradients on the horizontal axis, where different shades of grey show gene densities. The connecting bars, coloured from blue through green to red, denote syntenic orthologous loci identified by MCScanX, where the gradient of colour shows the *K*_a_/*K*_s_ values. Areas without connecting bars or with numerous crossings are regions lacking synteny either between haplotypes or between 2 species, referred to as nSRs.

We explored the conservation of gene order between haplotypes within the same species (Ref versus Alt) and homologous chromosomes between 2 species (*A. tenuis* Ref vs. *A. digitifera* Ref) and identified regions lacking conserved synteny, which we refer to as nSRs (Fig. 2, Figs. S7 and S8). The nSRs were not uniformly distributed along the chromosomes; instead, they were primarily situated towards the inner regions of the chromosomes. In contrast, synteny is typically preserved at both ends of all chromosomes. While the locations of nSRs are often consistent across the 2 species, some nSRs are present only in one species or show considerable variation in size between species. The proportion of nSRs varied among chromosomes, ranging from 15.9% for the alternate haplotype of chromosome 12 (chr12Alt) to 57.2% for the reference haplotype of chromosome 10 (chr10Ref) for *A. tenuis* (Fig. S9A, Table S5).

**Fig. 3.**
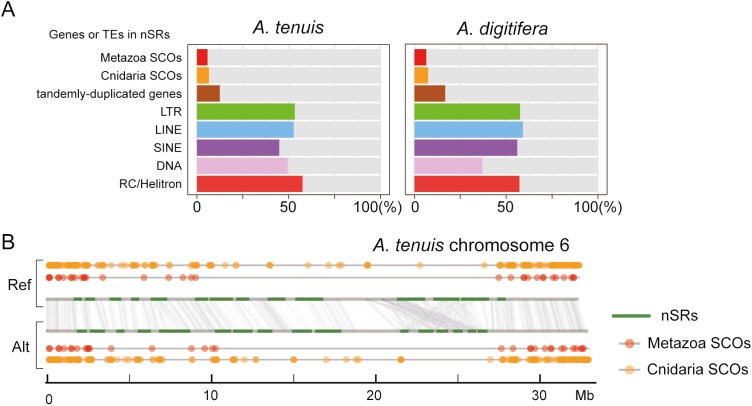
Biased distributions of SCOs and transposons in nSRs. (A) Gene and transposon contents in the coral genomes. The coloured bar charts indicate the proportion of genes/transposons located in nSRs. For instance, 1,794 BUSCO genes of Metazoa were identified in *Acropora tenuis* Ref and Alt genome assemblies in total. Among them, 105 BUSCO genes (5.8%) were located in nSRs. (B) A schematic diagram of chromosome 6 of *A. tenuis* serves as an example to depict the localization of SCOs, which are almost absent in nSRs. Upper and lower horizontal lines show reference and alternate haplotypes, respectively. The vertical lines in the middle depict homologous genes across 2 haplotypes and green horizontal lines indicate nSRs. Red and orange dots show the position of metazoan or cnidarian SCOs, respectively.

For the diploid genome assembly of *A. tenuis*, nSRs constituted 317.6 Mb out of 920.4 Mb total assembly, and 349.9 Mb out of 905.9Mb for *A. digitifera*. This means nSRs constitute 34.5% and 38.6% of their respective genomes (Figs. S9A and S10A, Tables S5 and S7). In the case of *A. tenuis*, 15,142 genes out of 54,389 total predicted gene models (27.8%) in Ref and Alt genomes were located in nSRs, which roughly corresponds to the proportion of nSR regions in the entire genome. Conversely, SCOs, which are present as a single copy in the genomes of different species, are rarely found in nSRs. In the Ref and Alt genome assemblies of *A. tenuis*, 1794 SCOs of metazoan species were identified using the BUSCO pipeline. Among them, only 105 genes (5.8%) were located in nSRs (Fig. 3A, Fig. S9B, Tables S5 and S6). Similarly, the distribution of SCOs of cnidarian animals (*A. tenuis*, *A. digitifera*, *N. vectensis*, and *H. magnipapillata*) identified using orthoMCL is also biased, with only 6.6% of SCOs being located in nSRs (Fig. 3A, Fig. S9C, Table S6). Using a hypergeometric test, we verified that the SCOs were scarcely present in the nSRs (*P* < 0.001, Table S6). Figure 3B shows an example of the biased distribution of SCOs that are almost exclusively absent in nSRs in chromosome 6 of *A. tenuis*. The same trend in the biased distribution of SCOs was also observed in the *A. digitifera* genome (Fig. 3A, Fig. S10B and C, Tables S7 and S8).

We further examined the distribution of species-specific, tandemly duplicated genes (SSTDGs) in coral genomes. SSTDG means that the tandem duplication occurred in only one species after speciation. Here, we defined a pair of genes as TDG when 2 genes are either adjacent to each other or arranged in tandem with one unrelated gene in between them. We identified 175 orthogroups, where one *Acropora* species has a single gene copy and another species has 2. The copy number is the same between haplotypes within the species, implying that the duplicated genes are already fixed in the species. Among the 56 SSTDGs in *A. tenuis*, 7 (12.5%) SSTDGs were located in nSRs (Fig. 3A, Table S6). Similarly, 20 out of 119 (16.8%) SSTDGs are found in the nSRs of *A. digitifera* (Fig. 3A, Table S8). Combined with the observations about the SCO distribution, we conclude that genes with fixed copy numbers tend to be located more in syntenic regions than in nSRs (Tables S6 and S8).

TEs were more abundant in nSRs than in syntenic regions (Fig. 3A, Figs. S7 and S8). For example, LTRs occupied 7.5% to 8.0% of *Acropora* genomes (Tables S9 and S10), and 53.4% of LTRs were located in nSRs in the *A. tenuis* genome. Similarly, TEs predominantly accumulated (more than 50%) in nSRs, except for SINE elements (44.9%) in *A. tenuis* and DNA transposons (37.1%) in *A. digitifera*. We surveyed enriched TEs in nSRs using a hypergeometric test (Tables S11 and S12). In *A. tenuis* genome, 14 types of DNA transposons, such as *Sola-3* and *Maverick*, and 6 types of retrotransposons, such as *BEL* LTR and *L1* LINE elements, were significantly enriched in nSRs (*q*-value < 0.001, Table S11). In case of *A. digitifera*, 19 types of DNA transposons and 7 types of retrotransposons were frequently distributed in nSRs (Table S12).

### Gene expansions in nSRs

We examined the enrichment of gene families with conserved functional domains in nSRs (Table 1, Tables S13 and S14). A large number of functional domains associated with TEs, such as reverse transcriptase and integrase, were detected. Excluding these, the most significantly enriched domains included the tetratricopeptide repeat (TPR), CHAT (Caspase HetF Associated with Tprs), glycosyl transferase group 1, and NACHT (NAIP, CIIA, HET-E, and TP1) (Table 1).

**Table 1.** Top 5 enriched Pfam domains in the non-syntenic regions in *Acroporatenuis* and *Acroporadigitifera* genomes.

Species	Pfam ID	Name	*q*-Value
*A. tenuis*	PF00078	Reverse transcriptase	7.39E−74
	PF17921	Integrase zinc binding domain	1.13E−72
	PF13424	Tetratricopeptide repeat	2.91E−60
	PF12770	CHAT domain	3.70E−60
	PF00534	Glycosyl transferases group 1	8.64E−60
*A. digitifera*	PF13424	Tetratricopeptide repeat	1.17E−97
	PF05729	NACHT domain	7.89E−85
	PF00534	Glycosyl transferases group 1	2.84E−84
	PF13176	Tetratricopeptide repeat	1.24E−80
	PF12770	CHAT domain	1.50E−76

**Fig. 4.**
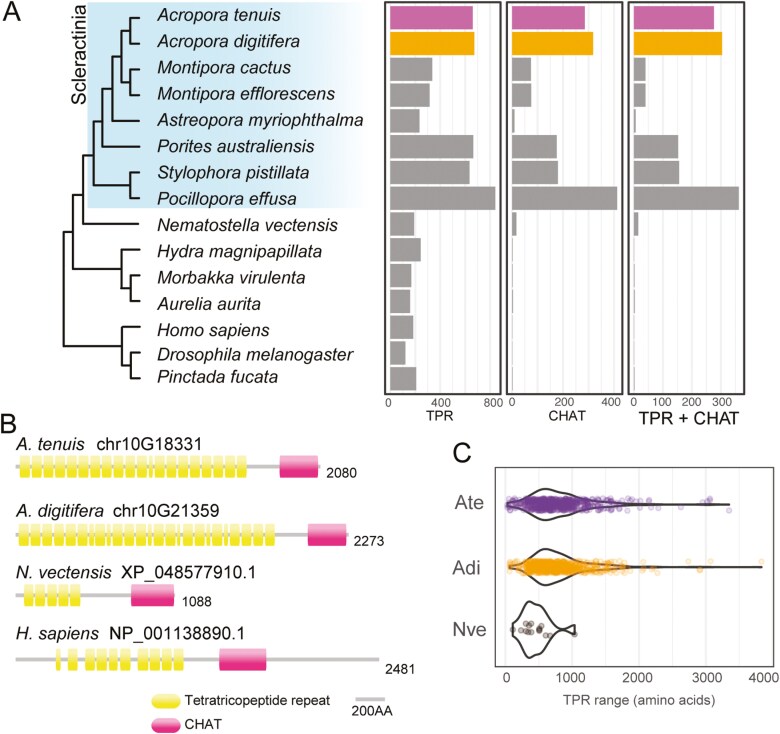
Gene family expansion and diversification of TPR-CHAT DCP genes in corals. (A) Genes encoding TPR and CHAT domain architecture are expanded in scleractinian corals after diversification from other cnidarians. (B) Examples of metazoan TPR-CHAT DCPs, demonstrating that the domain architecture of TPR-CHAT is conserved among cnidarians and human. (C) Length of TPR in TPR-CHAT DCPs are highly diverged in corals. Ate, *Acropora tenuis*; Adi, *Acropora digitifera*; Nve, *Nematostella vectensis*.

Genes encoding the CHAT domain (PfamID: PF12770) were remarkably duplicated in *Acropora* genomes (Fig. 4A). In most instances, the CHAT domain is encoded with the TPR (PF13176, PF13424) at the N-terminus (Fig. 4B). Hereafter, these gene products are designated as TPR-CHAT domain-containing proteins (DCPs). The *A. tenuis* and *A. digitifera* Ref haplotype genomes encode 276 and 304 TPR-CHAT DCPs, respectively, whereas the majority of metazoan species, except for anthozoans, have only 1 or 2 copies of the gene with the same domain architecture (Fig. 4A). The gene family expansion was reported in *Montipora* genomes.^25^ The gene number differed among coral species examined in this study, ranging from 6 (*Astreopora myriophthalma*) to 362 (*Pocillopora effusa*), and most of the coral genomes encoded more TPR-CHAT DCP genes than that of the sea anemone (*N. vectensis*, 15 genes). This suggests that extensive gene duplications of the TPR-CHAT family took place in the coral lineage after divergence from Actiniaria.

The domain architecture of TPR-CHAT, a long stretch of multiple TPR domains in the N-terminus followed by a single CHAT domain, is conserved among metazoans (Fig. 4B). The range of TPR sequences of TPR-CHAT DCPs of *Acropora* is significantly diverged compared to *N. vectensis* (Fig. 4C). The TPR sequences ranged from 31 to 3,347 amino acids (AAs) in *A. tenuis*, 63 to 3,832 AAs in *A. digitifera*, and 111 to 1036 in *N. vectensis*. The putative human homolog of TPR-CHAT is TTC28 or TPRBK (NP_001138890.1), which is expressed in various cell types and tissues to play a role in cytokinesis,^59^ although the precise functions of TPR and CHAT domains in TPRBK are not well understood. In general, the TPR motif is known to be involved in a wide range of biological functions, including molecular chaperoning, transcription, protein transport, and cell cycle regulation.^60,61^ Given that the TPR motif frequently serves as an interaction site for ligands, the presence of diverse TPR sequences may facilitate an increase in possible partner diversity.^61^ The notable variability in protein size and amino acid lengths in the TPR domain may provide functional diversity within the TPR-CHAT DCPs of corals.

**Fig. 5.**
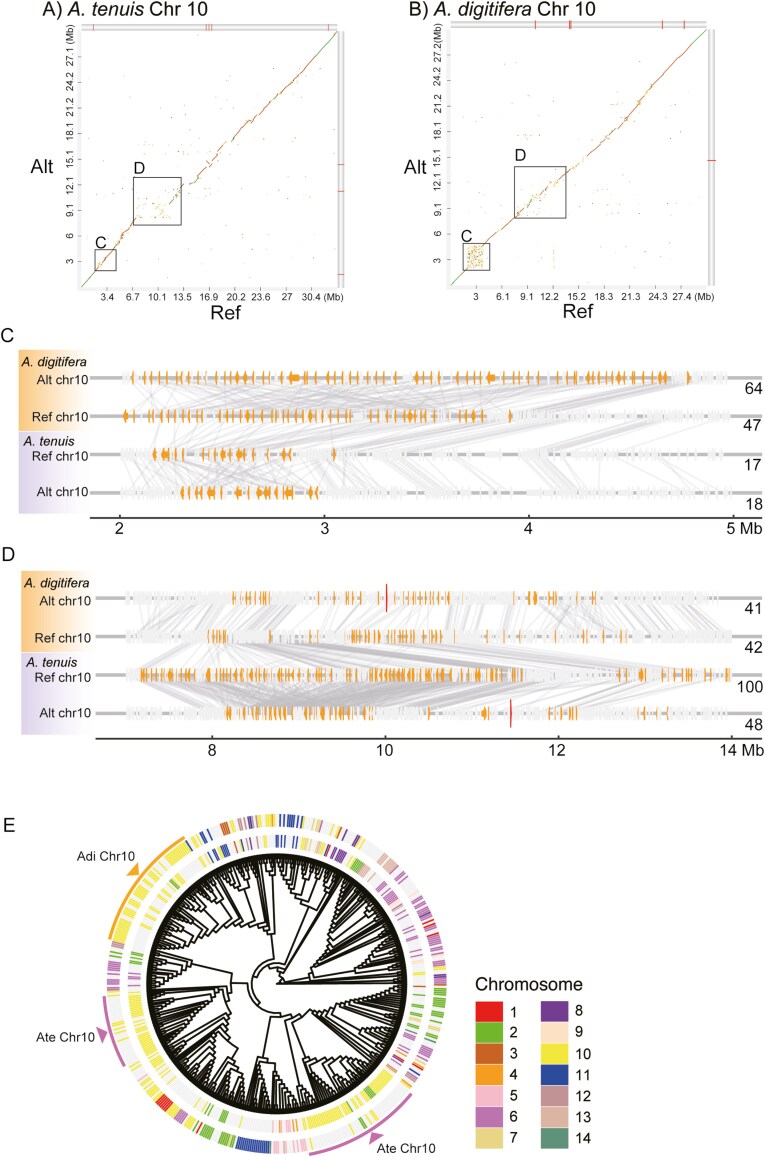
nSRs in chromosome 10 harbour TPR-CHAT DCP gene expansions. (A and B) Pairwise alignment of reference (Ref) and alternate (Alt) haplotypes of chromosome 10 of *Acropora tenuis* (A) and *Acropora digitifera* (B). nSRs lack the diagonal alignment, which is typically indicative of significant sequence similarity between homologous chromosomes. Red lines on the grey bars shown at the upper and right of the diagrams signify the gap locations of the scaffold. (C and D) Copy number variation of the TPR-CHAT DCP gene family at the nSRs on chromosome 10. (C) and (D) correspond to the areas enclosed by squares in (A) and (B), respectively. The orange boxes represent TPR-CHAT DCP genes. The numbers on the right side of the diagram indicate the count of TPR-CHAT DCP genes present in the displayed areas. Red vertical bars indicate gap positions of the scaffolds. Note that there are no assembly gaps in the nSRs, meaning that the nSRs and CNVs did not result from assembly errors. (E) Molecular phylogenetic tree of CHAT domain of TRP-CHAT DCPs from *A. tenuis* and *A. digitifera*. Outer and inner circles surrounding the phylogenetic tree indicate chromosome number of *A. tenuis* and *A. digitifera*, respectively. Note that TRP-CHAT DCP genes of *A. tenuis* (purple arc) and *A. digitifera* (orange arc) in chromosome 10 constitute different clusters, indicating that the gene family expansions have occurred in *A. tenuis* and *A. digitifera* independently.

Extensive tandem duplication of the TPR-CHAT DCP gene family was observed in nSRs (Fig. 5, Fig. S11). Taking chromosome 10 as an example, an nSR 2 to 5 Mbp from the scaffold’s 5′ terminus contains 47 (Ref) and 64 (Alt) TPR-CHAT DCP genes in the *A. digitifera* haplotypes, while only 17 or 18 TPR-CHAT DCP genes were present in corresponding genomic regions of the *A. tenuis* haplotypes (Fig. 5C). In *A. tenuis*, nSRs presented in different part of chromosome 10 (Fig. 5D) where 100 and 48 CHAT DCP genes were located in the Ref and Alt haplotypes, respectively. The molecular phylogenetic tree of the CHAT domains in these 2 coral genomes show that the duplicated genes in the 2 species are clustered separately (Fig. 5E). These results indicate that the gene copy number of the CHAT DCP gene increased through tandem duplication and that the duplication events occurred independently in the 2 coral genomes.

Genes encoding glycosyl transferase group 1 (PF00534), hereafter designated as glycos_transf_1 DCPs, also expanded in *Acropora* genomes (Table 1, Fig. S12). The glycos_transf_1 DCPs often contain repeat sequences such as TPR, an LRR (PF13516), and an Ankyrin repeat (PF00023) (Fig. S12A and B). The glycos_transf_1 DCPs with LRR often possess a NACHT domain in the middle part of the amino acid sequence, thus presenting a tripartite domain architecture similar to NLRs (nucleotide-binding domains and LRR-containing receptors).^7,62^ In contrast, most of the glycos_transf_1 genes with TPR or Ankyrin DCPs lack the NACHT domain (Fig. S12A). These glycos_transf_1 DCP genes were clustered in specific nSRs of *Acropora* chromosomes: glycos_transf_1 and TPR DCP genes in chromosome 11, glycos_transf_1 and LRR DCP genes in chromosome 5, and glycos_transf_1 and ankyrin DCP genes in chromosome 8, respectively (Fig. S12C). This indicates that glycos_transf_1 DCPs with different domain architectures were expanded by independent duplication events in each nSRs.

**Fig. 6.**
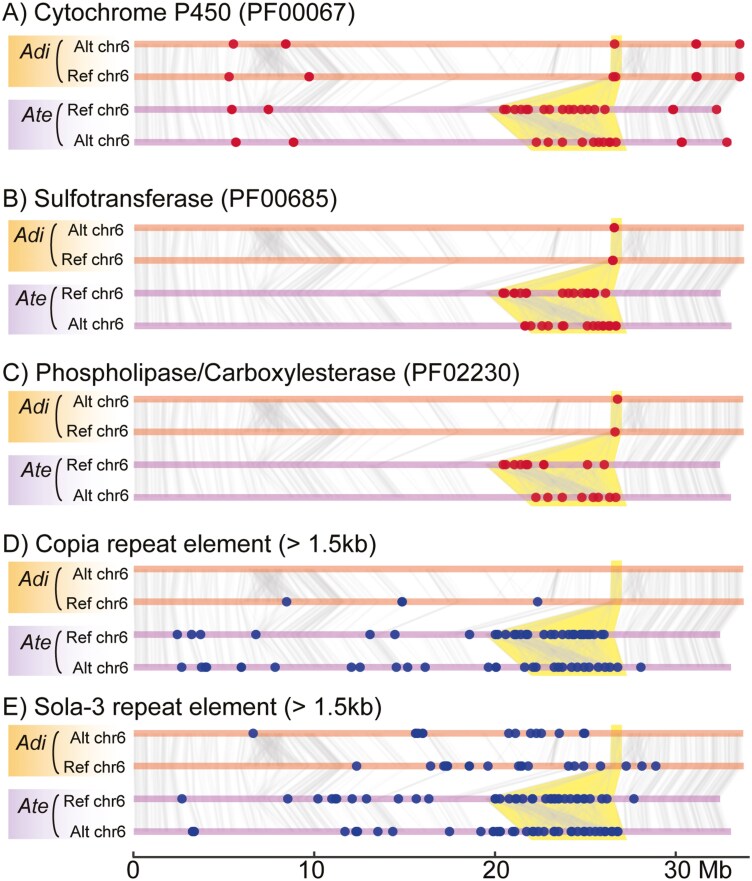
The gene duplications specific to chromosome 6 of *Acropora tenuis* encompass multiple gene families. Chromosome 6 of Ref and Alt haplotypes are represented by horizontal lines in orange (*Acropora digitifera*) and purple (*A. tenuis*). The vertical bars connect syntenic orthologous loci identified by MCScanX. Positions of genes encoding cytochrome P450 (A), sulfotransferase (B), and phospholipase/carboxylesterase (C) are shown in red circle, and positions of transposons *Copia* (D) and *Sola-3* (E) longer than 1.5 kb are shown in blue circle. Yellow shades indicate corresponding nSR among haplotypes.

A notable gene copy number variation between *A. tenuis* and *A. digitifera* was found in a nSR in chromosome 6 (Fig. 6). Copy numbers of genes encoding functional domains that include cytochrome P450 (PF00067), sulfotransferase (PF00685), and phospholipase/carboxylesterase (PF02230) were increased in the *A. tenuis* genome but not in the *A. digitifera* genome (Fig. 6A–C). In the corresponding genomic region of *A. digitifera* at this nSR location, there were only 1 to 4 copies per gene.

Furthermore, the nSR showed an elevation in the copy number of repeat elements such as the LTR transposon *Copia*^63^ and the DNA transposon *Sola-3*^64^ (Fig. 6D and E). These transposons are absent in the corresponding nSR of *A. digitifera*. The domain architecture of *Copia*, which is characterized by an integrase domain followed by a reverse transcriptase domain,^63^ was evident in Iso-seq reads (Fig. S13), implying that the *Copia* retrotransposon is expressed and can be translocated in the *A. tenuis* genome. Since *Copia* and *Sola-3* transposons were absent in the nSR of *A. digitifera* (Fig. 6D and E), these elements likely had been translocated to this region of *A. tenuis* genome after speciation of *A. tenuis* and *A. digitifera*.

**Fig. 7.**
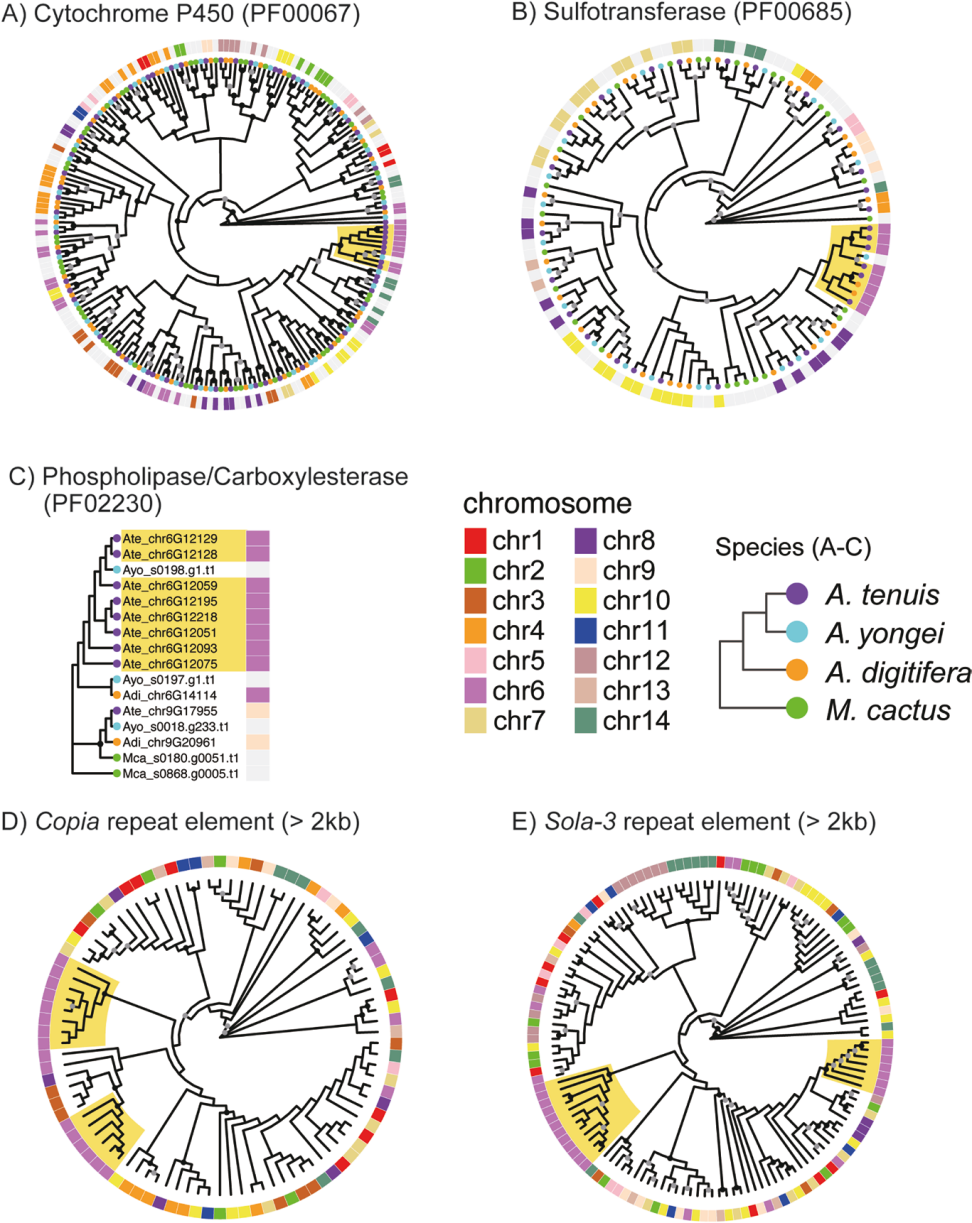
Molecular phylogenetic trees of gene families and transposons specifically expanded in *Acropora tenuis* genome. Outer circles surrounding the phylogenetic trees (A–E) and the box at the right of the tree (C) indicate chromosome numbers. Nodes of the tree supported with high bootstrap values are marked with black (=100 %) and grey dots (≥90%). Yellow shades indicate genes located in the nSR of chromosome 6 presented in Figure 6. (A–C) For cytochrome P450 domain, sequence longer that 350 amino acids (AAs) and longer than 500 AAs are used for the analysis. Similarly, >200 AAs and <350 AAs for sulfotransferase, and >150 AAs and <300 AAs for phospholipase/carboxylesterase. (D and E) For molecular phylogenies of *Copia* and *Sola-3* transposons, nucleotide sequences longer than 2 kb were used.

Molecular phylogenetic trees were constructed for functional domains of cytochrome P450, sulfotransferase, and phospholipase/carboxylesterase (Fig. 7A–C). Conserved domain sequences retrieved from *A. tenuis*, *A. digitifera*, *A. yongei,* and *M. cactus*^21^ were included in the analyses. Cytochrome P450 genes in the nSR in chromosome 6 of *A. tenuis* were clustered (Fig. 7A), indicating that these genes show a significant sequence similarity compared to the other family members located outside the nSR. Therefore, these clustered genes were likely derived from tandem duplication events within the nSRs. This is further evidenced by the molecular phylogenetic trees of sulfotransferase (Fig. 7B) and phospholipase/carboxylesterase (Fig. 7C). Notably, phylogenetic analysis of *Copia* and *Sola-3* transposons also showed that these elements of the nSR in chromosome 6 were clustered (Fig. 7D and E). As DNA transposons do not replicate their copy, copy number is not increased via the translocation process. So the increased copy number of transposons in this region also likely due to tandem duplication rather than their translocation activity. These observations indicate that cytochrome P450, sulfotransferase, and phospholipase/carboxylesterase and transposons have expanded through tandem duplications that happened simultaneously in the nSR.

**Fig. 8.**
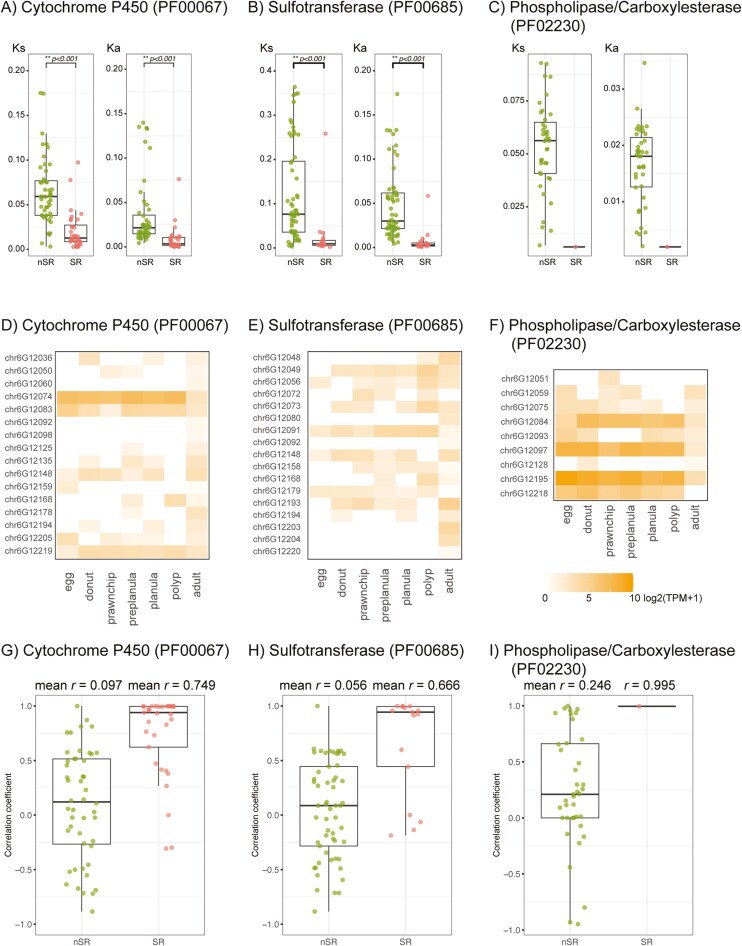
Nucleotide and expression diversity of gene families specifically expanded in *Acropora tenuis* chromosome 6. (A–C) *K*_s_ and *K*_a_ values between paralogs located in nSRs (green dots) and orthologs in syntenic regions (SRs, red dots). Significant differences of *K*_s_ and *K*_a_ between nSR and SR are performed by Student’s t-test. The statistical test was not conducted for phospholipase/carboxylesterase (C) because there is only 1 ortholog pair in SR. An outlier (*K*_s_ = 0.66, *K*_a_ = 0.22) in nSR of cytochrome P450 (A) was removed from the figure. (D–F) Gene expression profiles of expanded gene family in different developmental stages. (G–I) Pearson correlation coefficient (*r*) of gene expression pattern between paralogs located in nSRs (green dots) and orthologs in SRs (red dots). Mean values of *r* are shown in the upper part of the plot. For phospholipase/carboxylesterase, *r* between an orthologous pair in SR is shown (I).

To analyse the genetic diversity of cytochrome P450, sulfotransferase, and phospholipase/carboxylesterase, the synonymous and non-synonymous substitution rates among these homologous genes were estimated (Fig. 8A–C). Homologous gene pairs that occupy corresponding loci in the Ref and Alt haplotypes were determined by MCscanX, and *K*_a_ and *K*_s_ values between the pairs were calculated. If a gene pair is located in a syntenic region, it is considered to be orthologous. If a gene pair present in an nSR, these genes are highly likely paralogous. The majority of orthologous genes in syntenic regions showed low substitution rates (*K*_a_ < 0.02 and *K*_s_ < 0.05). On the other hand, homologous genes in nSRs showed higher substitution rates. For example, some of the duplicated sulfotransferase genes in the nSRs showed *K*_a_ > 0.1 and *K*_s_ = ~0.35 (Fig. 8B). Mean *K*_s_ values were significantly higher than *K*_a_. These high substitution rates of gene families demonstrate that the gene expansion in nSRs allow relaxed selective pressure to increase genetic diversity between gene copies.

Mutations in regulatory regions of duplicated genes can alter their expression patterns, leading to the gain of new functions (neofunctionalization) or the division of a gene’s original roles (subfunctionalization).^65^ To examine the functional diversification of these expanded gene families in an nSR, RNA-seq of developmental stages, including unfertilized egg, donut, prawnchip, preplanula, planula, polyp, and adult tissues, were analysed (Fig. 8D–F). Almost all duplicated genes were expressed in the adult, while varied expression patterns were observed through developmental stages before the polyp stage.

We further investigated the correlation of gene expression patterns between homologous genes in Ref and Alt haplotypes identified by McScanX (Fig. 8G–I). The Pearson correlation coefficient (*r*) values of expanded gene family members varied in nSRs. The mean correlation coefficient for cytochrome P450, sulfotransferase, and phospholipase/carboxylesterase families in nSRs is 0.097, 0.056, and 0.246, respectively (Fig. 8G–I). The Pearson correlation coefficient close to zero suggests that there is little to no correlation in the expression patterns between the homologous gene pairs in nSRs. In contrast, the expression patterns show strong positive correlations in syntenic regions (mean *r* = 0.749, 0.666, and 0.995, respectively, in the gene families), indicating that the orthologous gene pairs in the Ref and Alt haplotypes are functionally conserved.

## Discussion

Recent advances in large-scale genome sequencing across a diverse array of metazoan taxa have uncovered the frequent occurrence of gene expansion events. These findings have significant implications for our understanding of evolutionary processes and the mechanisms underlying genetic diversity. While the phenomenon of gene expansion is well-documented across a variety of taxa, the mechanisms driving this process remain incompletely understood. In this study, by comparing the genomes of 2 closely related coral species, we discovered evidence of species-specific gene expansion. Furthermore, we precisely separated 2 haplotypes from a single individual, which represent the haplotypic variation within the population. By comparing these 2 haplotypes, we succeeded in capturing the dynamic changes in the genome occurring within the population.

It has generally been believed that the genetic information is nearly identical and that synteny is completely conserved in paired haplotypes. The differences between haplotypes have been considered to be limited to local variations such as single-nucleotide polymorphisms and SVs. The present study demonstrated that *Acropora* corals possess genomic regions where synteny is highly disrupted. The nSRs are characterized by the expansion of diverse gene families. The discovery of gene expansion in nSRs is the second case, the first being our study of the haplotype-phased genome of the pearl oyster *P. fucata*.^6^ Given its identification in 2 taxonomically distant groups, suggests this phenomenon could be prevalent among various organisms. Notably, NLR gene clustering in chromosomal regions with large-scale SV has been reported in zebrafish.^8^ It is not clear whether the NLR gene family expansion occurred in nSRs of zebrafish genome because in the study, the haplotype variation was detected by sequence alignment to the reference rather than via a haplotype-phasing approach. It would be fascinating to test the presence of nSRs in fish genomes by producing haplotype-phased genome assemblies thereof.

Comparison of the genomes of *A. tenuis* and *A. digitifera* reveals that nSRs are often situated on homologous genomic areas (Fig. 2). Since gene expansion of the same gene families is found in corresponding nSRs, these nSRs may have emerged prior to the speciation of the 2 species. Molecular dating analysis of *Acropora* species indicates that the divergence time of *A. tenuis* and *A. digitifera* is ~52 million years ago,^21^ suggesting the nSRs have been maintained for at least 50 million years in the coral genomes. Therefore, the presence of nSRs may not be a transient state of pre-fixed duplicating genes but rather a state maintained in population for a long time.

High-quality, haplotype-phased genomes enabled the comprehensive identification of species-specific nSRs and gene family expansion events. The species-specific gene expansion events may not be detected by gene count comparisons. For instance, the gene number of the CHAT DCP family increases in both *A. tenuis* and *A. digitifera*, leading to an interpretation that the gene expansion occurred in their common ancestor. On the contrary, our whole genome syntenic comparison and molecular phylogenies indicate that the CHAT DCP family expansion has occurred not only in their common ancestor but also in each species independently (Fig. 5). This result highlights the remarkable flexibility of genomes, allowing copy number variation of gene families. nSRs serve as a platform for the expansion and diversification of gene families.

In *Acropora* genomes, gene expansion was observed in gene families with a broad spectrum of functions. Genes encoding functional domains such as CHAT, glycosyl transferase group 1, and NACHT, combined with repeat domains including TPR, LRR, and Ankyrin repeats, are expanded in coral genomes (Fig. 5, Figs. S11 and S12). The expansion of gene families involved in immune responses illustrates a critical aspect of evolutionary adaptation. The NLR gene family has expanded to include a large number of alleles, each capable of handling different pathogens. This diversity is crucial for the immune system’s ability to recognize and respond to a broad spectrum of infectious agents, thereby increasing the organism’s survival chances in pathogen-rich environments.

Cytochrome P450, which is believed to be ubiquitously present across all metazoans, consists of enzymes that metabolize a variety of substrates, including endogenous molecules and xenobiotics.^66,67^ The cytochrome P450 family contributes to detoxification processes by catalysing the oxidation of exogenous molecules. This oxidation enhances the reactivity and hydrophilicity of these molecules, facilitating their removal from the body.^68,69^ Sulfotransferase enzymes conjugate sulfuryl groups to low-molecular-weight exogenous compounds. This sulfonation has a detoxifying effect on the substrate by inactivating it and increasing its hydrophilicity.^70^ The expansion of these gene families may benefit the organism’s survival by increasing the quantity of gene products and enhancing the capacity to interact with diverse ligands.

One notable phenomenon observed in chromosome 6 of the *A. tenuis* genome is that different gene families, including cytochrome P450, sulfotransferase, and phospholipase/carboxylesterase, are expanding simultaneously within the same nSR (Fig. 6). These gene families appear to be functionally unrelated. When a set of genes located in close genomic proximity lacks constraints against copy number increase, they can be duplicated simultaneously by genetic hitchhiking^71^ along with the tandem duplication of a gene family under positive selection. Indeed, transposons have also expanded within the same nSR, providing solid evidence of tandem duplication of DNA sequences in a neutral state through hitchhiking.

**Fig. 9.**
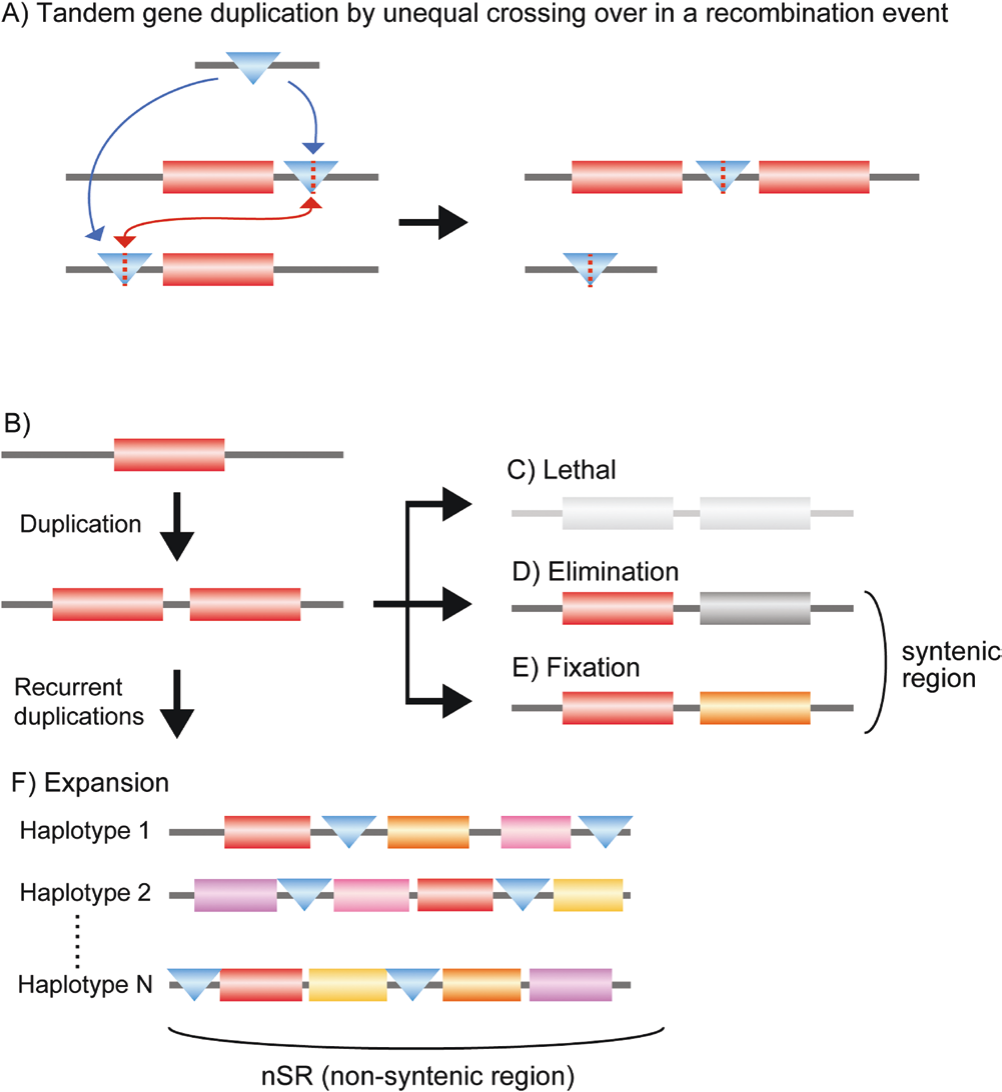
Model of gene tandem duplication and its subsequent fate. (A) Tandem duplication by unequal crossing over during a recombination event. If repetitive sequences, such as transposons, are introduced at the 5′ and 3′ ends of a gene, unequal crossing over can produce a duplicated gene in 1 haplotype and result in the loss of the gene locus in another. (B–F) Evolutionary fate of duplicate genes. (C) If the gene duplication is lethal, the haplotype will be eliminated from the population. (D and E) Under neutral conditions, the duplicated copy may become a pseudogene (D) or be fixed (E) by random genetic drift. These genes with consistent copy numbers between haplotypes are typically found in syntenic regions. (F) Tandem duplication can occur multiple times in a specific region where an increase in gene copy number is neutral or adaptive. The duplicated sequence can serve as a source for further duplications, accelerating recurrent duplication events in nSRs. Boxes with different colours indicate alleles, and triangles represent transposons.

The increase in gene copy number and the rearrangement of genes observed in the nSRs can be explained by the non-allelic homologous recombination (NAHR) model.^72^ NAHR occurs either through unequal crossing over or via the break-induced replication (BIR) pathway. Unequal crossing over happens during meiosis when homologous chromosomes misalign and exchange non-equivalent segments, resulting in the tandem duplication of genetic material in 1 haplotype and a corresponding deletion in the other (Fig. 9A). BIR is a DNA repair mechanism that responds to double-strand breaks in replicating DNA.^73^ When a replication fork is damaged, the broken molecule may use an ectopic sequence as a template to resume replication. This process can result in the generation of DNA with duplications, deletions, and inversions. It is possible that NAHR is facilitated by the presence of similar nucleotide sequences, such as transposons, located in proximal genomic regions. For example, if a transposon is inserted at the 5′ position of a gene in 1 haplotype and at the 3′ position in another haplotype, unequal crossing over can occur between the transposon sites, resulting in tandem duplication of the gene in 1 haplotype and loss of the gene in the other (Fig. 9A). Furthermore, the duplicated DNA sequences can trigger subsequent NAHR events, accelerating recurrent duplications that lead to increased gene copy numbers and structural changes in the region.

Tandem duplication is a stochastic event that can occur in any region of the genome. Nevertheless, regions experiencing large-scale gene family expansion are restricted and are clustered in nSRs. The fate of duplicated genes is considered to be linked to the impact of the gene copy number changes on the adaptive fitness of the organism^74,75^ (Fig. 9B–F). Gene expansion is not invariably beneficial for all genes. Increased gene copy numbers can alter protein levels, thereby disrupting the balance of precise gene expression networks. If an increase in specific gene products causes fatal dosage imbalances, the haplotype carrying duplicated genes will be quickly eliminated from the population (Fig. 9C). BUSCO genes serve as a typical example of those under strong selective pressure to avoid duplication in the haploid genome. In coral genomes, BUSCO genes are predominantly found outside nSRs (Fig. 3, Figs. S9 and S10, Tables S6 and S8), indicating that these loci are under strong negative selection pressure against gene duplication. When duplication is either neutral or has only a slight impact on fitness, the gene copy may become a pseudogene or be retained by genetic drift (Fig. 9D and E). Such fixed states are typically found in syntenic regions.

Gene duplication can occur multiple times in the same region if it is adaptive, for example, by increasing dosage and/or accumulating beneficial mutations in gene copies. Gene family expansion can also occur randomly under neutral conditions,^76^ serving as preadaptation. Recurrent gene duplications and diversification may be advantageous for gene families involved in immunity and biological defence, as an expanded gene repertoire enhances broad reactivity against ligands. Furthermore, a diploid individual can harbour doubled gene repertoires in terms of copy number and combinations in nSRs across 2 haplotypes. In fact, expanded gene families of cytochrome P450, sulfotransferase, and phospholipase/carboxylesterase in *A. tenuis* genome showed various gene expression patterns among paralogs (Fig. 8D–I), indicating their functional diversification. Diverged gene repertoires between haplotypes can enhance tolerance to external stimuli and environmental fluctuations, potentially providing advantages at both the population and individual levels.

In a previous study, we discussed that recombination cannot occur in nSRs due to their SVs between haplotypes, resulting in reduced genome diversity in offspring.^6^ However, non-homologous recombination may still occur between nSRs as they contain partial sequence similarities (eg, transposons and paralogous gene copies). In this scenario, gene repertoires and copy numbers in nSRs could be mixed during meiosis, generating haplotype diversity among offspring. This effect is more pronounced in organisms with high fecundity, thereby enhancing the survival chances of offspring. In order to examine the hypothesis that NAHR has occurred in nSRs, further study is essential to compare the genomes before and after the recombination event, specifically the parental and the offspring genomes.

## Conclusion

The nearly T2T, haplotype-phased assembly of 2 coral genomes allows for the identification of nSRs and comprehensive research into lineage-specific gene family expansions. These nSRs serve as platforms for the expansion and diversification of gene families. They contribute to the increase in gene families, such as those related to biological defence, where an expanded gene repertoire enhances the organism’s adaptive capabilities. In particular, the expansion of coral genes associated with innate immunity and stress response is crucial for their symbiosis with dinoflagellates and resilience to environmental challenges. The presence of nSRs in phylogenetically unrelated species, such as coral and bivalve genomes, suggests that nSRs are prevalent and play a pivotal role in evolution and adaptation. This hypothesis will be tested through the further accumulation of haplotype-phased genome assembly data across diverse metazoan taxa.

## Supplementary Material

dsaf017_suppl_Supplementary_Materials

## Data Availability

All PacBio and Illumina reads are available in DDBJ and can be accessed with DRR accession numbers DRR608901 to DRR608909. Genome assemblies and predicted gene models are available at https://marinegenomics.oist.jp/gallery.
