# Supplementary material for: Nearly T2T, phased genome assemblies of corals reveal haplotype diversity and the evolutionary process of gene expansion
Source: DNA Res. 2025 Jul 1;32(4):dsaf017. doi: 10.1093/dnares/dsaf017 (PMC12314368; doi:10.1093/dnares/dsaf017)
Supplement: dsaf017_suppl_Supplementary_Materials [file dsaf017_suppl_supplementary_materials.pdf]

Nearly T2T, phased genome assemblies of corals reveal haplotype diversity and the evolutionary process of gene expansion

Takeshi Takeuchi, Yoshihiko Suzuki, Eiichi Shoguchi, Manabu Fujie, Mayumi Kawamitsu, Chuya Shinzato Noriyuki Satoh, and Eugene W. Myers

Supplemental Materials

Contents

1. Supplementary Tables S1–S14

2. Supplementary Figures S1-S13

1. Supplementary Tables

Supplementary Table S1. Summary of *Acropora* genome sequence data.

Assembly	Sequencer	Library type	Number of bases(Gbp)	Number of reads (million)	Average read length (bp)	Estimated coverage depth*
A. tenuis	PacBio Sequel II	HiFi SMRTbell	19.4	0.76	25,379	42.2
	Illumina HiSeqX	Omni-C	40.5	135.1 x2	150	88.0
A. digitifera	PacBio Sequel II	HiFI SMRTbell	38.4	1.66	23,126	83.5
	Illumina HiSeqX	Omni-C	136.5	453.6 x2	150	296.7

* Genome size of 460 Mb for both species

Supplementary Table S2. Summary of *Acropora* genome assemblies.

	A. tenuis			*A. digitifera*		
	All	Haplotype Ref	Haplotype Alt	All	Haplotype Ref	Haplotype Alt
Number of contigs	277	75	82	314	90	108
contig N50 length (bp)	12,354,314	13,560,935	11,486,920	8,519,996	8,519,996	8,650,645
Number of scaffolds	148	14	14	144	14	14
scaffold N50 length (bp)	33,034,005	33,544,513	32,321,049	32,988,518	33,064,550	32,507,267
Total length (bp)	929,860,843	462,350,242	458,077,591	916,955,618	454,925,515	451,022,489
Sum of chromosomal scaffolds (bp)	920,427,833	462,350,242	458,077,591	905,948,004	454,925,515	451,022,489
Number of Gaps	129	61	68	170	76	94
Reliable block N50 (Mb)	11.5	14.9	12.5	8.0	9.3	7.4
k-mer QV	68.6	69.6	69	68.6	68.8	69.8
k-mer completeness	99.0	77.6	77.4	98.9	73.1	72.5
BUSCO benchmarking (metazoa_odb10)						
Complete (%)	93.0	94.4	94.7	94.2	94.6	94.4
Complete and single copy (%)	8.1	93.9	94.1	11.7	94.0	94.0
Complete and duplicated (%)	84.9	0.5	0.6	82.5	0.6	0.4
Fragmented (%)	1.4	1.3	1.4	0.5	0.9	1.4
Missing (%)	5.6	4.3	3.9	5.3	4.5	4.2

Supplementary Table S3. Sequence lengths and length differences between haplotypes of *A. tenuis* genome assembly.

Reference Haplotype				Alternate Haplotype				Length difference (bp)
scaffold ID	Telomere	Number of gaps	Length (bp)	scaffold ID	Telomere	Number of gaps	Length (bp)	
chr1Ref	Both ends	4	42,260,570	chr1Alt	No	2	42,175,515	85,055
chr2Ref	Both ends	1	40,596,019	chr2Alt	One end	3	40,631,120	35,101
chr3Ref	No	1	35,084,758	chr3Alt	No	2	35,241,107	156,349
chr4Ref	Both ends	3	36,097,252	chr4Alt	Both ends	3	33,819,035	2,278,217
chr5Ref	Both ends	1	33,544,513	chr5Alt	Both ends	3	33,102,502	442,011
chr6Ref	Both ends	2	32,437,148	chr6Alt	Both ends	3	33,034,005	596,857
chr7Ref	Both ends	12	32,316,035	chr7Alt	One end	7	32,321,049	5,014
chr8Ref	Both ends	8	31,271,468	chr8Alt	One end	6	32,209,985	938,517
chr9Ref	Both ends	5	33,707,779	chr9Alt	Both ends	9	30,979,219	2,728,560
chr10Ref	Both ends	5	33,741,717	chr10Alt	One end	3	30,235,115	3,506,602
chr11Ref	Both ends	7	27,896,473	chr11Alt	No	11	30,444,069	2,547,596
chr12Ref	Both ends	3	28,194,345	chr12Alt	One end	5	27,682,130	512,215
chr13Ref	Both ends	5	27,399,128	chr13Alt	Both ends	7	29,157,566	1,758,438
chr14Ref	Both ends	4	27,803,037	chr14Alt	Both ends	4	27,045,174	757,863

Supplementary Table S4. Sequence lengths and length differences between haplotypes of *A. digitifera* genome assembly.

Reference Haplotype				Alternate Haplotype				Length difference (bp)
Scaffold ID	Telomere	Number of gaps	Length (bp)	Scaffold ID	Telomere	Number of gaps	Length (bp)	
chr1Ref	No	5	39,045,714	chr1Alt	One end	9	37,986,678	1,059,036
chr2Ref	No	8	39,786,426	chr2Alt	One end	10	38,759,970	1,026,456
chr3Ref	Both ends	6	33,304,500	chr3Alt	Both ends	6	34,703,315	1,398,815
chr4Ref	One end	5	33,368,340	chr4Alt	One end	8	34,390,035	1,021,695
chr5Ref	Both ends	7	33,739,456	chr5Alt	One end	3	32,507,267	1,232,189
chr6Ref	Both ends	3	33,737,820	chr6Alt	Both ends	4	33,714,464	23,356
chr7Ref	Both ends	10	31,511,376	chr7Alt	No	7	32,988,518	1,477,142
chr8Ref	Both ends	4	31,841,008	chr8Alt	One end	5	30,949,241	891,767
chr9Ref	Both ends	8	33,064,550	chr9Alt	Both ends	8	29,212,585	3,851,965
chr10Ref	One end	1	30,424,130	chr10Alt	One end	5	30,244,026	180,104
chr11Ref	Both ends	5	31,358,363	chr11Alt	Both ends	5	30,934,994	423,369
chr12Ref	Both ends	6	29,923,323	chr12Alt	Both ends	6	29,220,142	703,181
chr13Ref	Both ends	4	27,485,174	chr13Alt	One end	11	27,871,415	386,241
chr14Ref	Both ends	4	26,335,335	chr14Alt	Both ends	7	27,539,839	1,204,504

Supplementary Table S5. Lengths of non-syntenic regions of *A. tenuis* genome assembly.

Scaffold ID	Chromosome	nSR length	nSR%	number of BUSCO in nSR	total number of BUSCO	BUSCO% in nSR
chr1Ref	42,260,570	19,200,000	45.43%	1	51	2.0%
chr2Ref	40,596,019	10,300,000	25.37%	11	106	10.4%
chr3Ref	35,084,758	5,900,000	16.82%	3	83	3.6%
chr4Ref	36,097,252	8,100,000	22.44%	6	92	6.5%
chr5Ref	33,544,513	12,200,000	36.37%	4	51	7.8%
chr6Ref	32,437,148	14,600,000	45.01%	2	33	6.1%
chr7Ref	32,316,035	12,700,000	39.30%	2	40	5.0%
chr8Ref	31,271,468	12,200,000	39.01%	6	64	9.4%
chr9Ref	33,707,779	9,500,000	28.18%	1	83	1.2%
chr10Ref	33,741,717	19,300,000	57.20%	4	38	10.5%
chr11Ref	27,896,473	12,000,000	43.02%	1	42	2.4%
chr12Ref	28,194,345	5,100,000	18.09%	3	70	4.3%
chr13Ref	27,399,128	6,400,000	23.36%	5	69	7.2%
chr14Ref	27,803,037	9,700,000	34.89%	3	77	3.9%
Total	462,350,242	157,200,000	34.00%	52	899	5.8%
Scaffold ID	Chromosome	nSR length	nSR%	number of BUSCO in nSR	total number of BUSCO	BUSCO% in nSR
chr1Alt	42,175,515	22,400,000	53.1%	1	53	1.89%
chr2Alt	40,631,120	9,700,000	23.9%	8	106	7.55%
chr3Alt	35,241,107	6,900,000	19.6%	1	78	1.28%
chr4Alt	33,819,035	5,600,000	16.6%	5	95	5.26%
chr5Alt	33,102,502	11,600,000	35.0%	2	51	3.92%
chr6Alt	33,034,005	14,400,000	43.6%	4	33	12.12%
chr7Alt	32,321,049	16,700,000	51.7%	5	40	12.50%
chr8Alt	32,209,985	13,200,000	41.0%	6	63	9.52%
chr9Alt	30,979,219	7,800,000	25.2%	2	82	2.44%
chr10Alt	30,235,115	16,800,000	55.6%	6	35	17.14%
chr11Alt	30,444,069	14,600,000	48.0%	3	43	6.98%
chr12Alt	27,682,130	4,400,000	15.9%	1	68	1.47%
chr13Alt	29,157,566	7,100,000	24.4%	4	69	5.80%
chr14Alt	27,045,174	9,200,000	34.0%	5	79	6.33%
Total	458,077,591	160,400,000	35.0%	53	895	5.92%

Supplementary Table S6. Single copy orthologs (SCOs) and tandemly duplicated genes are less abundant in the non-syntenic regions in *A. tenuis* genome.

Gene category	Number of genes in nSRs	Number of genes in whole genome	p-value*
All predicted gene models	15142	54389	-
Metazoan SCOs	105	1794	0.00.E+00
Cnidarian SCOs	642	9706	0.00.E+00
Tandemly-duplicated genes	7	56	1.70.E-03

* *p*-value of hypergeometric test

Supplementary Table S7. Lengths of non-syntenic regions of *A. digitifera* genome assembly.

Scaffold ID	Chromosome	nSR length	nSR%	number of BUSCO in nSR	total number of BUSCO	BUSCO% in nSR
chr1Ref	39,045,714	24,200,000	61.98%	2	53	3.77%
chr2Ref	39,786,426	13,600,000	34.18%	10	104	9.62%
chr3Ref	33,304,500	4,100,000	12.31%	2	79	2.53%
chr4Ref	33,368,340	5,100,000	15.28%	3	87	3.45%
chr5Ref	33,739,456	12,900,000	38.23%	5	50	10.00%
chr6Ref	33,737,820	22,100,000	65.51%	1	32	3.13%
chr7Ref	31,511,376	17,200,000	54.58%	5	38	13.16%
chr8Ref	31,841,008	12,000,000	37.69%	3	59	5.08%
chr9Ref	33,064,550	11,500,000	34.78%	2	80	2.50%
chr10Ref	30,424,130	20,000,000	65.74%	6	37	16.22%
chr11Ref	31,358,363	18,200,000	58.04%	4	42	9.52%
chr12Ref	29,923,323	6,300,000	21.05%	3	67	4.48%
chr13Ref	27,485,174	6,600,000	24.01%	4	68	5.88%
chr14Ref	26,335,335	5,900,000	22.40%	4	75	5.33%
Total	454,925,515	179,700,000	39.50%	54	871	6.20%
Scaffold ID	Chromosome	nSR length	nSR%	number of BUSCO in nSR	total number of BUSCO	BUSCO% in nSR
chr1Alt	37,986,678	23,700,000	62.39%	2	52	3.85%
chr2Alt	38,759,970	10,600,000	27.35%	12	104	11.54%
chr3Alt	34,703,315	4,200,000	12.10%	2	82	2.44%
chr4Alt	34,390,035	6,900,000	20.06%	5	92	5.43%
chr5Alt	32,507,267	11,500,000	35.38%	6	51	11.76%
chr6Alt	33,714,464	20,900,000	61.99%	1	33	3.03%
chr7Alt	32,988,518	19,300,000	58.51%	2	36	5.56%
chr8Alt	30,949,241	12,100,000	39.10%	6	62	9.68%
chr9Alt	29,212,585	7,000,000	23.96%	5	81	6.17%
chr10Alt	30,244,026	20,600,000	68.11%	6	37	16.22%
chr11Alt	30,934,994	16,100,000	52.04%	4	43	9.30%
chr12Alt	29,220,142	4,200,000	14.37%	0	70	0.00%
chr13Alt	27,871,415	7,200,000	25.83%	4	70	5.71%
chr14Alt	27,539,839	5,900,000	21.42%	5	73	6.85%
Total	451,022,489	170,200,000	37.74%	60	886	6.77%

Supplementary Table S8. Single copy orthologs (SCOs) and tandemly duplicated genes are less abundant in the non-syntenic regions in *A. digitifera* genome.

Gene category	Number of genes in vSRs	Number of genes in whole genome	p-value*
All predicted gene models	22969	62661	-
Metazoan SCOs	114	1757	1.60.E-199
Cnidarian SCOs	728	8978	0.00.E+00
Tandemly-duplicated genes	20	119	5.45.E-07

* *p*-value of hypergeometric test

Supplementary Table S9. Repeat elements in *A. tenuis* genome.

Retrotransposons (Class I)		
LTR	Gypsy	3.21%
	Pao	0.80%
	DIRS	0.71%
	Ngaro	0.65%
	Copia	0.47%
	BEL	0.36%
	ERV	0.07%
	ERVK	0.04%
	ERV1	0.04%
	ERVL	0.01%
	unclassified	1.66%
	total	8.00%
LINE	Penelope	3.20%
	L2	2.02%
	L1	1.25%
	Dong	0.49%
	CR1	0.38%
	Rex	0.33%
	CRE	0.27%
	RTE	0.25%
	I	0.05%
	R2	0.03%
	unclassified	0.15%
	total	8.41%
SINE	tRNA	0.84%
	MIR	0.09%
	ID	0.04%
	total	0.97%
DNA transposons (Class II)		
	TcMar	4.84%
	hAT	4.78%
	nMITE	3.50%
	MITE	1.89%
	PIF	1.35%
	Academ	0.54%
	Sola	0.52%
	Crypton	0.48%
	Helitron	0.44%
	CMC	0.44%
	Harbinger	0.37%
	Maverick	0.29%
	Mutator	0.26%
	Kolobok	0.25%
	P	0.16%
	IS3EU	0.15%
	MULE	0.12%
	CACTA	0.12%
	Merlin	0.07%
	Ginger	0.04%
	Zator	0.04%
	Novosib	0.02%
	PiggyBac	0.01%
	unclassified	2.67%
	total	23.33%
unknown		
	total	6.50%
Tandem repeats		
	microsatellite (1-6bp)	0.36%
	7-10bp	0.11%
	minisatellite (11-100bp)	1.05%
	satellite (>100bp)	0.50%
	total	2.01%

Supplementary Table S10. Repeat elements in *A. digitifera* genome.

Retrotransposons (Class I)		
LTR	Gypsy	2.94%
	Pao	0.84%
	DIRS	0.52%
	Ngaro	0.46%
	BEL	0.30%
	ERVK	0.26%
	Copia	0.23%
	ERV1	0.15%
	ERV	0.11%
	unclassified	1.65%
	total	7.46%
LINE	Penelope	3.33%
	L2	1.97%
	L1	1.07%
	Dong	0.36%
	Rex	0.33%
	CR1	0.27%
	RTE	0.20%
	CRE	0.16%
	I	0.01%
	R2	0.01%
	unclassified	0.09%
	total	7.78%
SINE	tRNA	0.94%
	MIR	0.23%
	U	0.02%
	ID	0.01%
	B4	0.01%
	Alu	0.00%
	unclassified	0.07%
	total	1.29%
DNA transposons (Class II)		
	TcMar	7.61%
	hAT	4.43%
	MITE	3.52%
	nMITE	3.08%
	PIF	1.21%
	Sola	0.63%
	Mutator	0.60%
	Academ	0.60%
	Harbinger	0.53%
	Helitron	0.41%
	Crypton	0.37%
	CMC	0.25%
	Kolobok	0.22%
	Maverick	0.18%
	MULE	0.17%
	P	0.12%
	Merlin	0.11%
	IS3EU	0.11%
	Ginger	0.08%
	PiggyBac	0.06%
	CACTA	0.05%
	Dada	0.01%
	Zator	0.01%
	Zisupton	0.01%
	unclassified	2.54%
	total	26.91%
unknown		
	total	5.63%
Tandem repeats		
	microsatellite (1-6bp)	0.33%
	7-10bp	0.07%
	minisatellite (11-100bp)	0.87%
	satellite (>100bp)	0.63%
	total	1.91%

Supplementary Table S11. Enriched transposable elements in the non-syntenic regions in *A. tenuis* genome.

TE Order/Superfamily	q-value*
Retrotransposons (Class I)	
LTR/BEL	0.000509566
LINE/L1-Tx1	0.004370361
LINE/RTE	0.004757411
LINE/Penelope	0.005266888
LINE/L1	0.005791896
SINE/tRNA-Core	3.51E-08
DNA transposons (Class II)	
DNA/Sola-3	1.22E-27
DNA/Maverick	6.97E-15
DNA/nMITE	2.61E-07
DNA/TcMar-Tc2	4.51E-07
DNA/Kolobok-T2	2.52E-06
DNA/TcMar-Pogo	9.26E-06
DNA/MULE-MuDR	1.26E-05
DNA/PIF-ISL2EU	0.000868289
DNA/IS3EU	0.000892088
DNA/hAT-hAT5	0.001706033
DNA/PIF-Harbinger	0.002922331
DNA/hAT	0.004606314
DNA/Academ-H	0.009282091
DNA/Academ-2	0.009971024

*Adjusted *q*-value of hypergeometric test

Supplementary Table S12. Enriched transposable elements in the non-syntenic regions in *A. digitifera* genome.

TE Order/Superfamily	q-value*
Retrotransposons (Class I)	
LTR/ERVK	1.12E-07
LTR/Pao	6.54E-05
LTR/DIRS	7.59E-05
LINE/RTE-BovB	2.50E-10
LINE/L1-Tx1	0.003700989
SINE/tRNA	5.00E-21
SINE/MIR	0.008752166
DNA transposons (Class II)	
DNA/nMITE	2.61E-50
DNA/MULE-MuDR	3.17E-28
DNA/Sola-3	3.45E-22
DNA/hAT	1.29E-15
DNA/CMC-EnSpm	3.34E-15
DNA/PIF-Harbinger	3.90E-15
DNA/TcMar-Tc2	3.96E-14
DNA/Maverick	4.02E-14
DNA/Merlin	1.47E-10
DNA/Crypton-V	1.36E-05
DNA/hAT-hAT1	0.000107535
DNA/Crypton-A	0.000122108
DNA/Sola-1	0.0008341
DNA/Academ-2	0.001079157
DNA/Ginger-2	0.001494994
DNA/TcMar-Pogo	0.001823513
DNA/CACTA	0.003644332
DNA/PiggyBac	0.003758934
DNA/Kolobok-T2	0.004418655

*Adjusted *q*-value of hypergeometric test

Supplementary Table S13. Enriched Pfam domains in the non-syntenic regions in *A. tenuis* genome.

Pfam ID	Name	q-value*
PF00078	Reverse transcriptase (RNA-dependent DNA polymerase)	7.39E-74
PF17921	Integrase zinc binding domain	1.13E-72
PF13424	Tetratricopeptide repeat	2.91E-60
PF12770	CHAT domain	3.70E-60
PF00534	Glycosyl transferases group 1	8.64E-60
PF00665	Integrase core domain	3.52E-53
PF13176	Tetratricopeptide repeat	2.24E-50
PF05380	Pao retrotransposon peptidase	4.31E-46
PF13359	DDE superfamily endonuclease	1.18E-42
PF17919	RNase H-like domain found in reverse transcriptase	7.70E-39
PF05729	NACHT domain	1.89E-38
PF09588	YqaJ-like viral recombinase domain	4.15E-34
PF05485	THAP domain	7.58E-34
PF03564	Protein of unknown function (DUF1759)	5.76E-29
PF18701	Family of unknown function (DUF5641)	1.15E-28
PF00098	Zinc knuckle	2.26E-23
PF14291	Domain of unknown function (DUF4371)	1.50E-22
PF05699	hAT family C-terminal dimerisation region	1.53E-22
PF17917	RNase H-like domain found in reverse transcriptase	6.62E-22
PF18738	DZIP3/ hRUL138-like HEPN	6.31E-21
PF14529	Endonuclease-reverse transcriptase	1.07E-19
PF13613	Helix-turn-helix of DDE superfamily endonuclease	4.41E-17
PF03184	DDE superfamily endonuclease	6.83E-17
PF13837	Myb/SANT-like DNA-binding domain	2.00E-15
PF02992	Transposase family tnp2	3.96E-15
PF13927	Immunoglobulin domain	4.70E-14
PF05585	Putative peptidase (DUF1758)	4.80E-14
PF08205	CD80-like C2-set immunoglobulin domain	5.15E-14
PF13843	Transposase IS4	5.11E-12
PF13151	Protein of unknown function (DUF3990)	8.03E-12
PF13516	Leucine Rich repeat	3.46E-11
PF13895	Immunoglobulin domain	7.09E-11
PF13692	Glycosyl transferases group 1	7.31E-11

*Adjusted *q*-value of hypergeometric test

Supplementary Table S14. Enriched Pfam domains in the non-syntenic regions in *A. digitifera* genome.

Pfam ID	Name	q-value*
PF13424	Tetratricopeptide repeat	1.17E-97
PF05729	NACHT domain	7.89E-85
PF00534	Glycosyl transferases group 1	2.84E-84
PF13176	Tetratricopeptide repeat	1.24E-80
PF12770	CHAT domain	1.50E-76
PF17921	Integrase zinc binding domain	1.56E-66
PF13359	DDE superfamily endonuclease	5.88E-64
PF05380	Pao retrotransposon peptidase	6.18E-60
PF00078	Reverse transcriptase (RNA-dependent DNA polymerase)	4.81E-54
PF18701	Family of unknown function (DUF5641)	1.29E-46
PF00098	Zinc knuckle	1.29E-41
PF05485	THAP domain	4.70E-39
PF09588	YqaJ-like viral recombinase domain	1.04E-38
PF13613	Helix-turn-helix of DDE superfamily endonuclease	1.07E-37
PF03564	Protein of unknown function (DUF1759)	2.56E-29
PF00791	ZU5 domain	8.37E-28
PF17919	RNase H-like domain found in reverse transcriptase	5.93E-27
PF00665	Integrase core domain	5.23E-22
PF05699	hAT family C-terminal dimerisation region	1.30E-20
PF14291	Domain of unknown function (DUF4371)	3.24E-20
PF17917	RNase H-like domain found in reverse transcriptase	6.49E-20
PF03184	DDE superfamily endonuclease	1.66E-18
PF13516	Leucine Rich repeat	1.78E-16
PF13843	Transposase IS4	5.29E-16
PF02992	Transposase family tnp2	2.23E-15
PF03221	Tc5 transposase DNA-binding domain	3.48E-13
PF08797	HIRAN domain	1.58E-11

*Adjusted *q*-value of hypergeometric test

2. Supplementary Figures

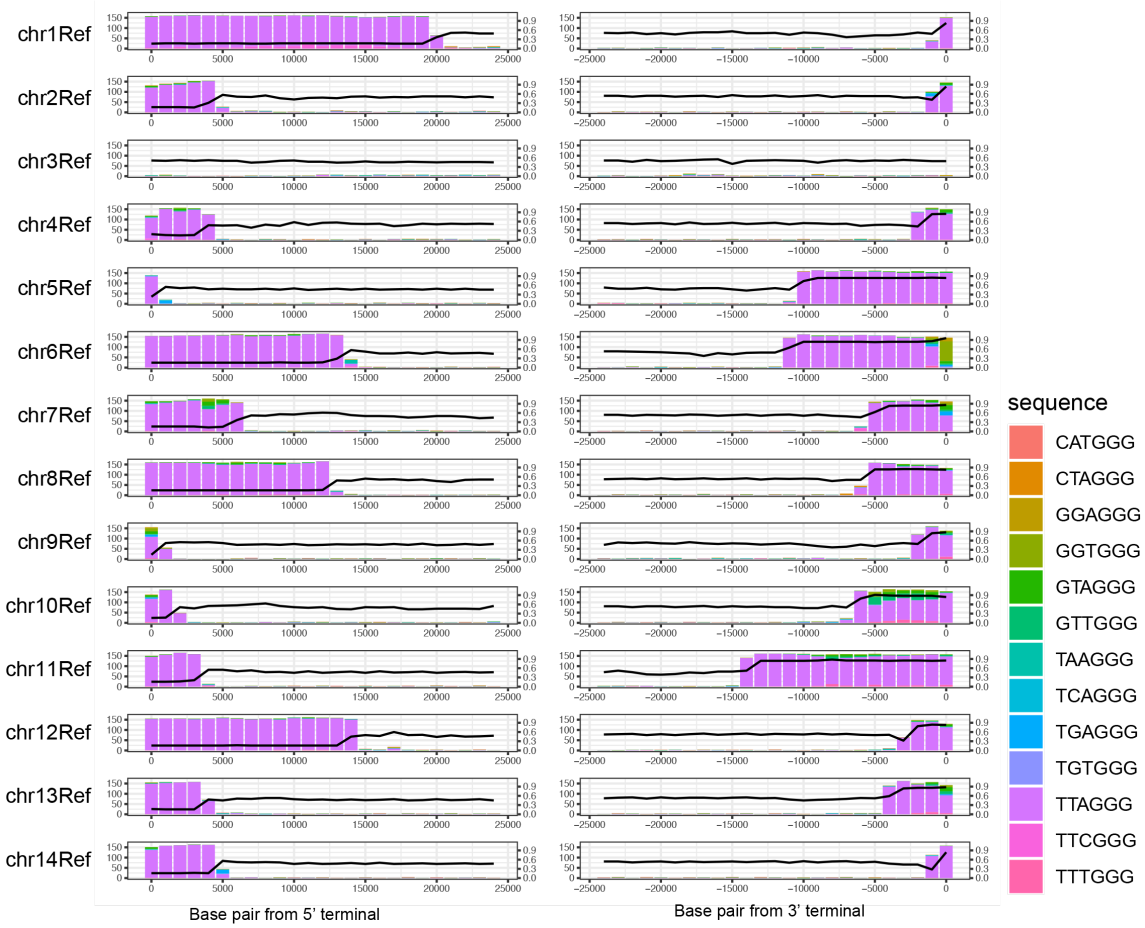

Supplementary Figure S1. Telomere sequences in *A. tenuis* genome assembly reference haplotype. Bar plots indicate the amount of the sequence motif, while line plots show the percentage of G and T nucleotides.

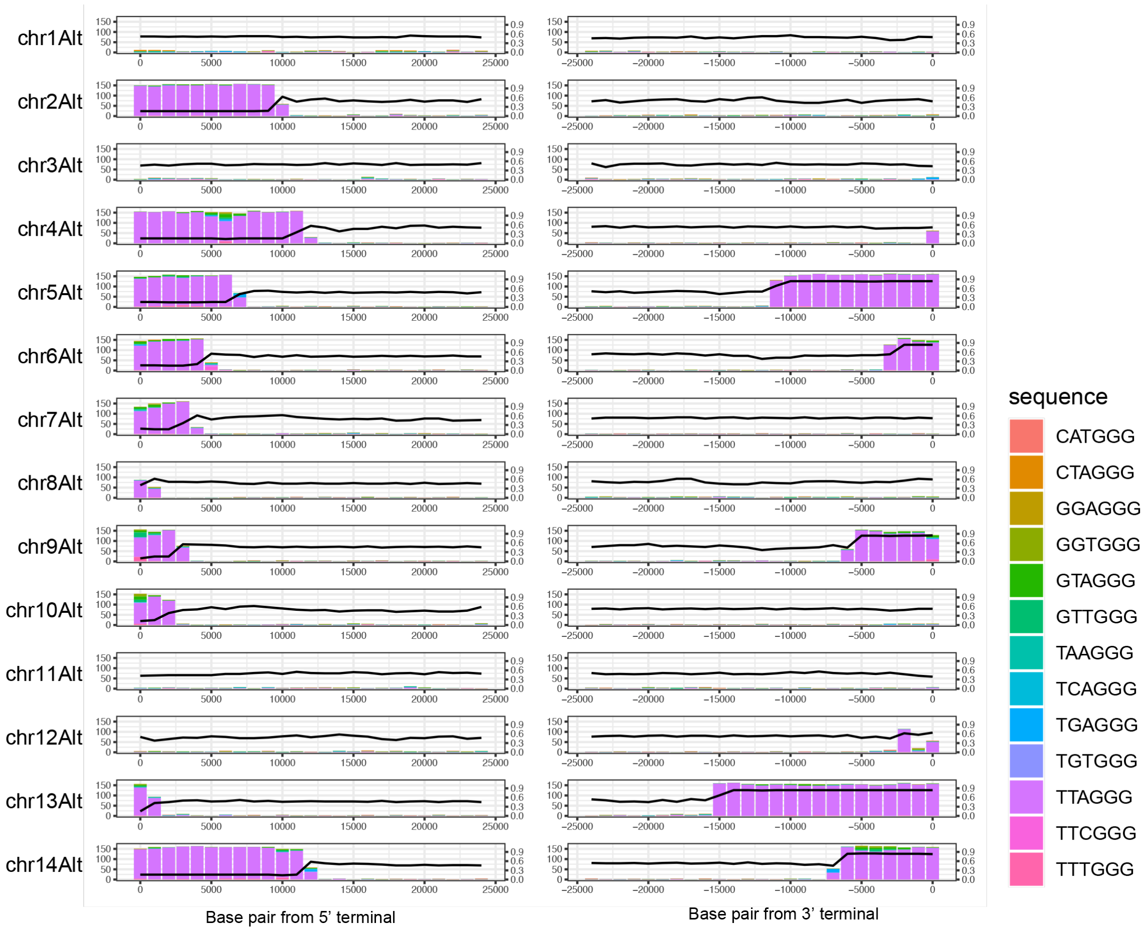

Supplementary Figure S2. Telomere sequences in *A. tenuis* genome assembly alternate haplotype. Bar plots indicate the amount of the sequence motif, while line plots show the percentage of G and T nucleotides.

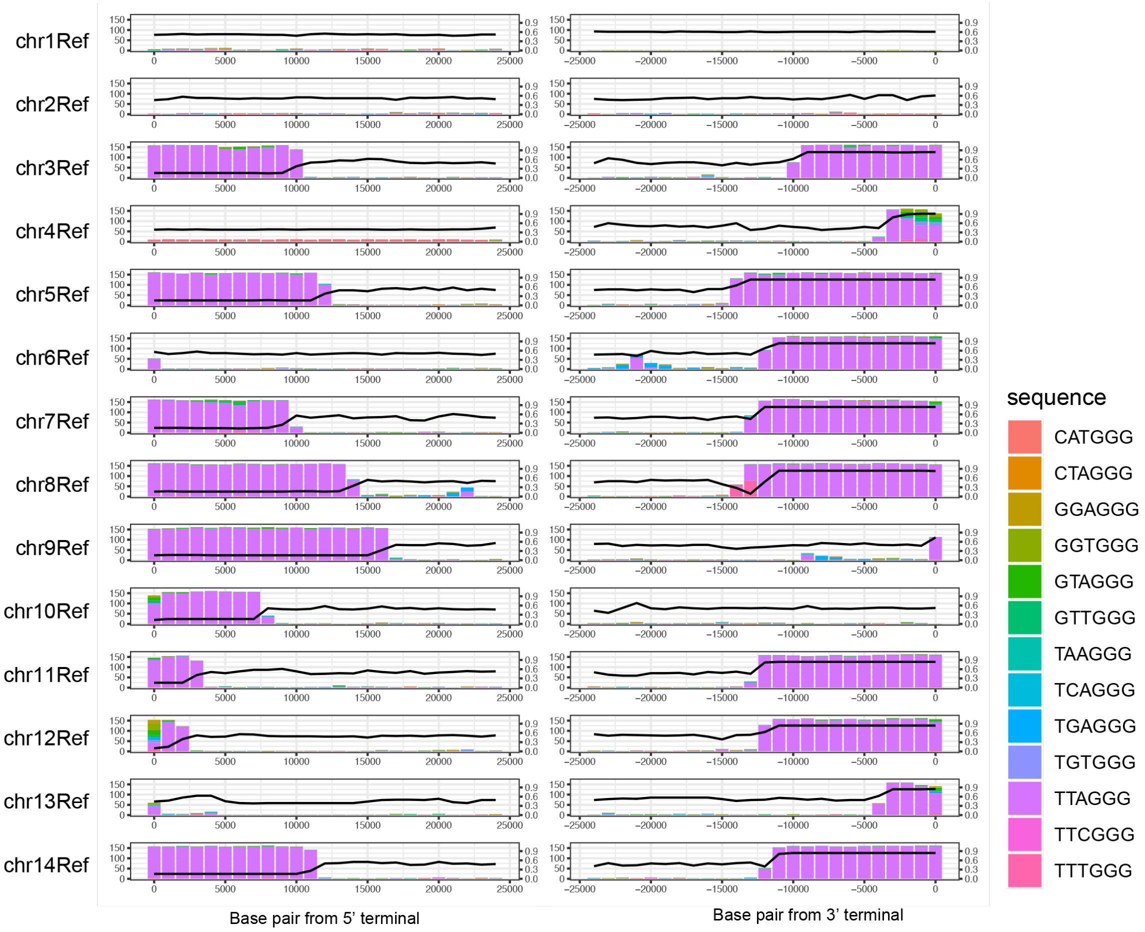

Supplementary Figure S3. Telomere sequences in *A. digitifera* genome assembly reference haplotype. Bar plots indicate the amount of the sequence motif, while line plots show the percentage of G and T nucleotides.

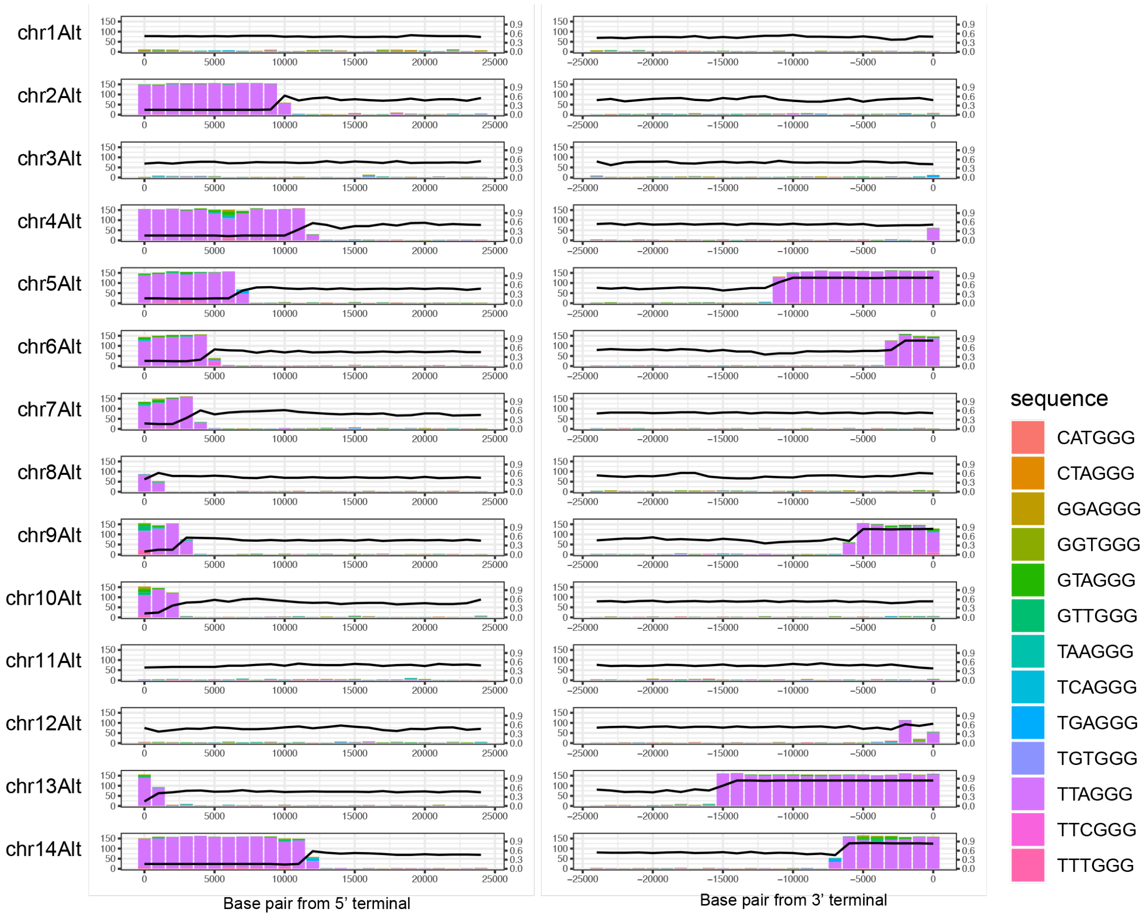

Supplementary Figure S4. Telomere sequences in *A. digitifera* genome assembly alternate haplotype. Bar plots indicate the amount of the sequence motif, while line plots show the percentage of G and T nucleotides.

Supplementary Figure S5

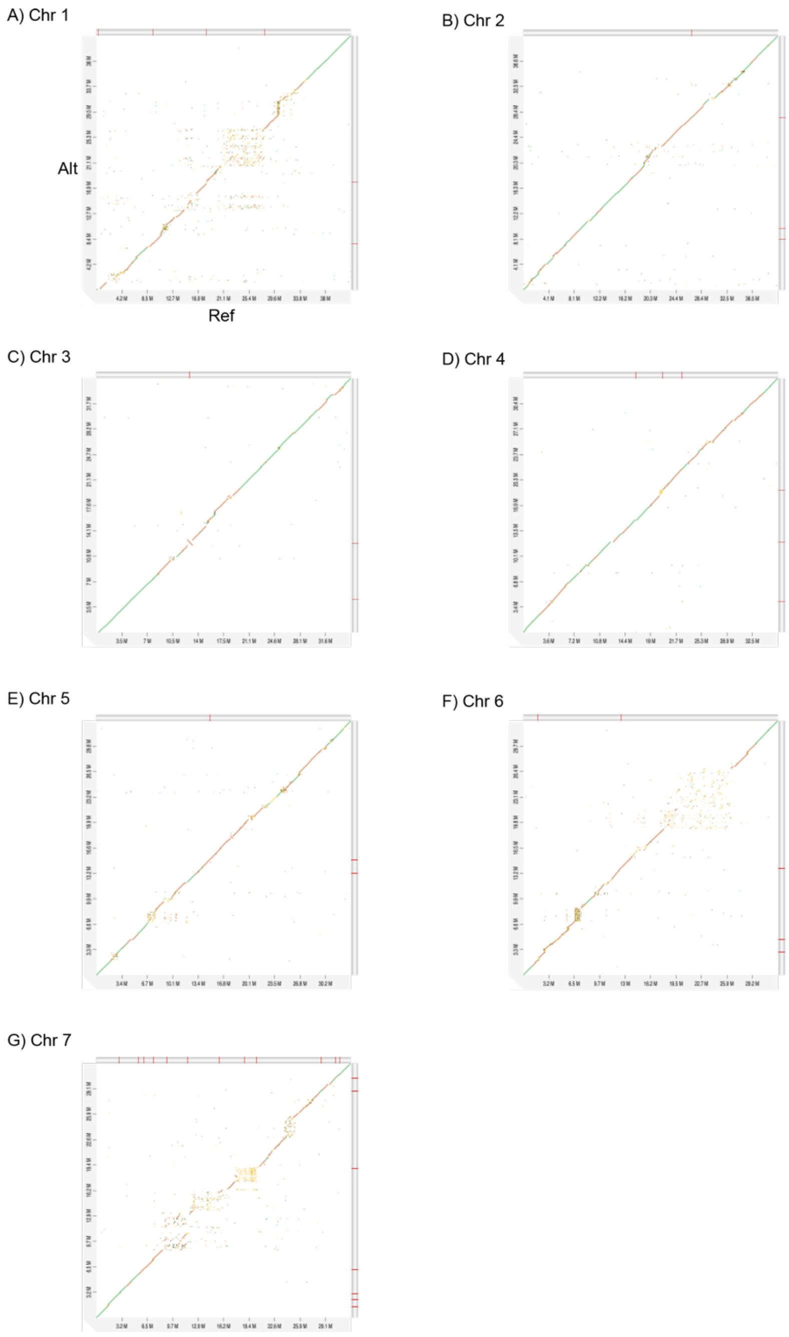

Supplementary Figure S5 (Continued)

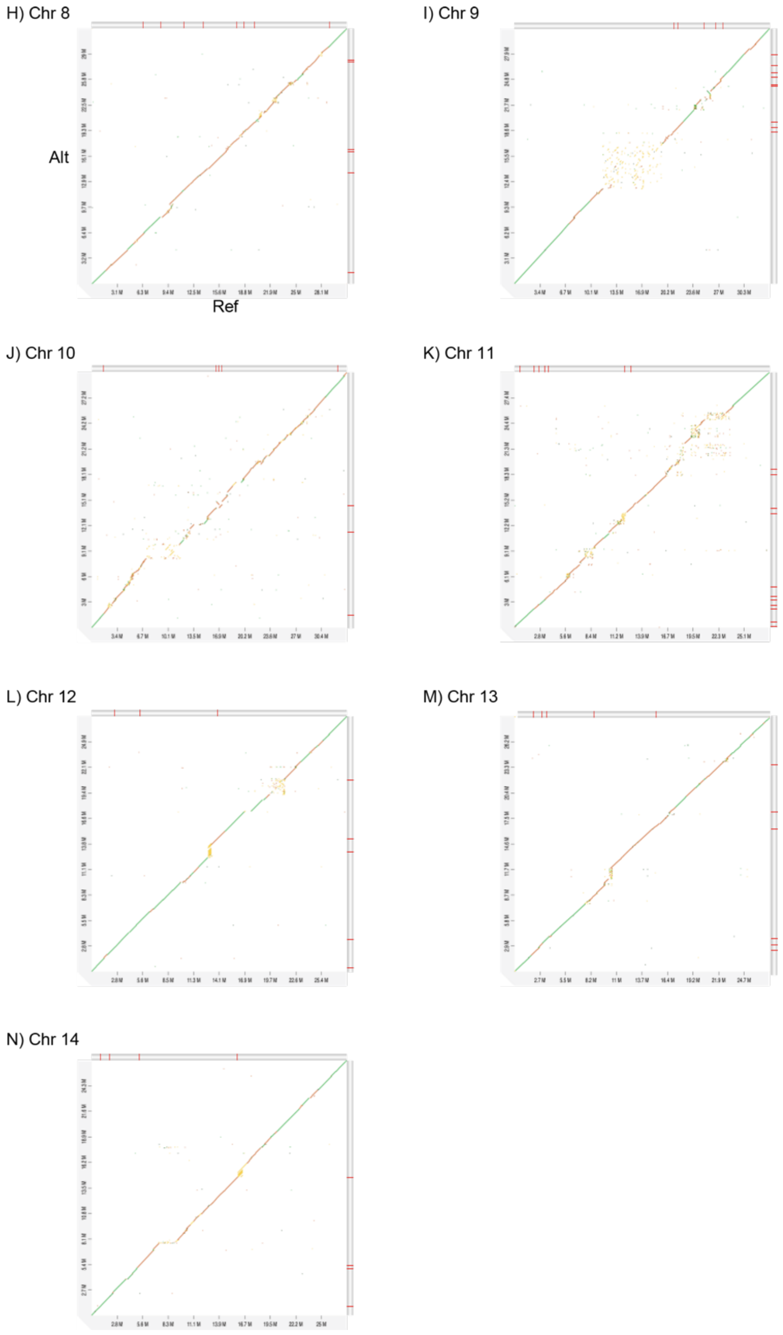

Supplementary Figure S5. Pairwise alignments of homologous chromosomes in reference (Ref) and alternate (Alt) haplotypes of *A. tenuis*. Alignment dot plots were generated using d-genies. Aligned segments are represented as red (forward alignment) or blue (reverse alignment) dots. Red lines on gray bars at the top and right of the dot plots indicate gap positions in the scaffolds.

Supplementary Figure S6

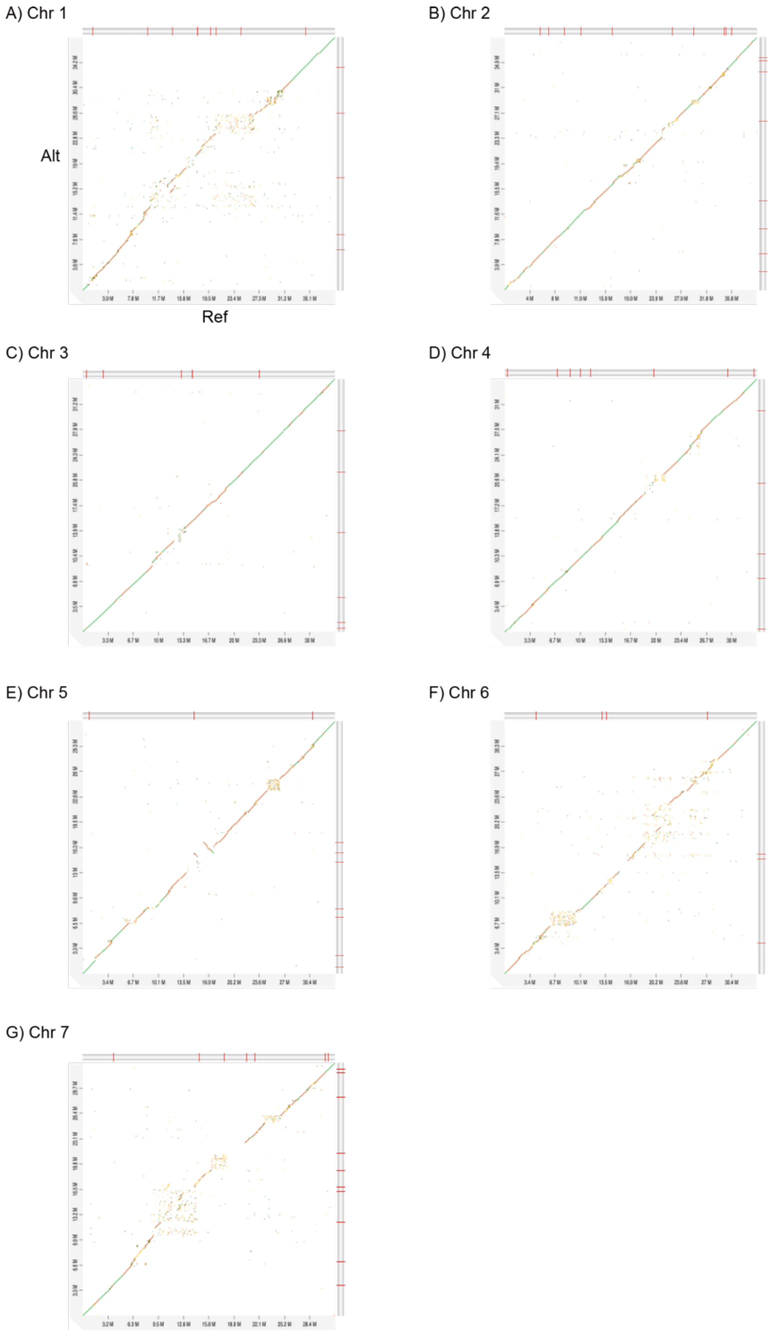

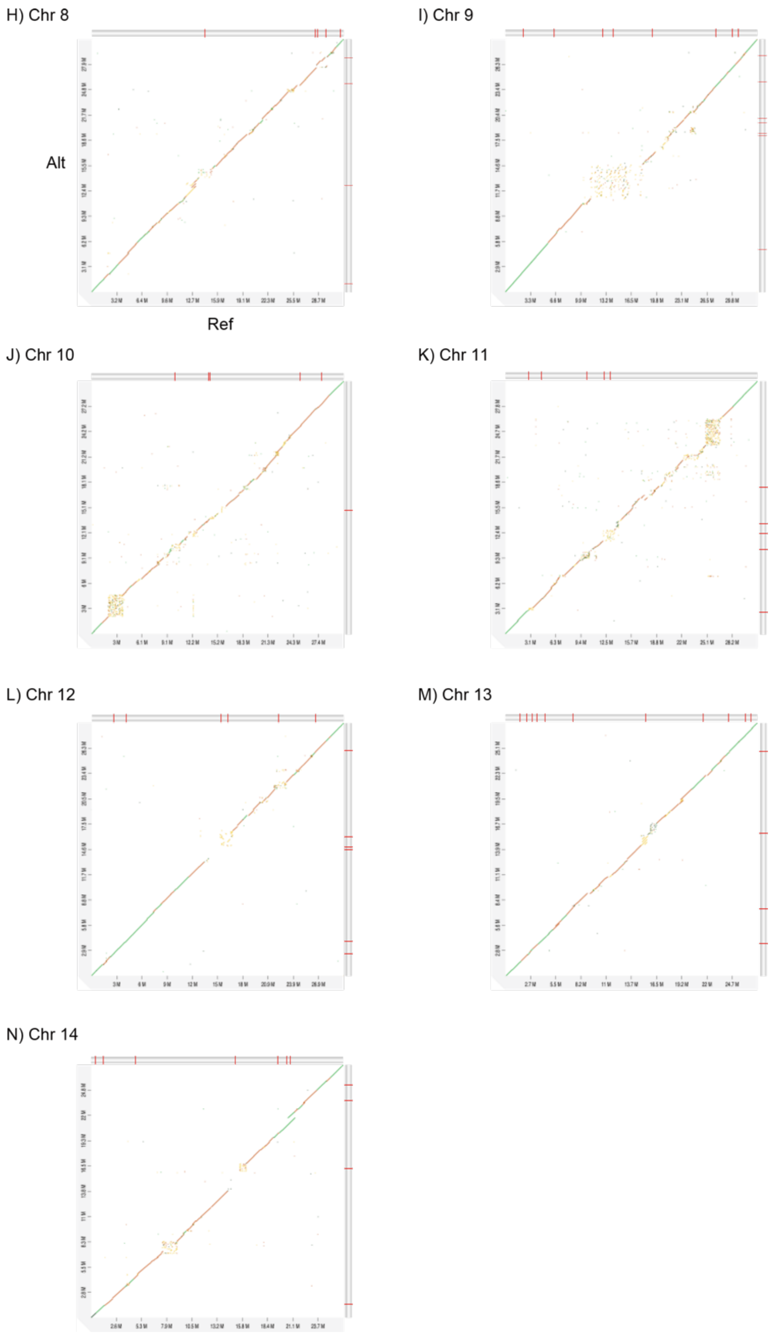

Supplementary Figure S6. Pairwise alignments of homologous chromosomes in reference (Ref) and alternate (Alt) haplotypes of *A. digitifera*. Alignment dot plots were generated using d-genies. Aligned segments are represented as red (forward alignment) or blue (reverse alignment) dots. Red lines on gray bars at the top and right of the dot plots indicate gap positions in the scaffolds.

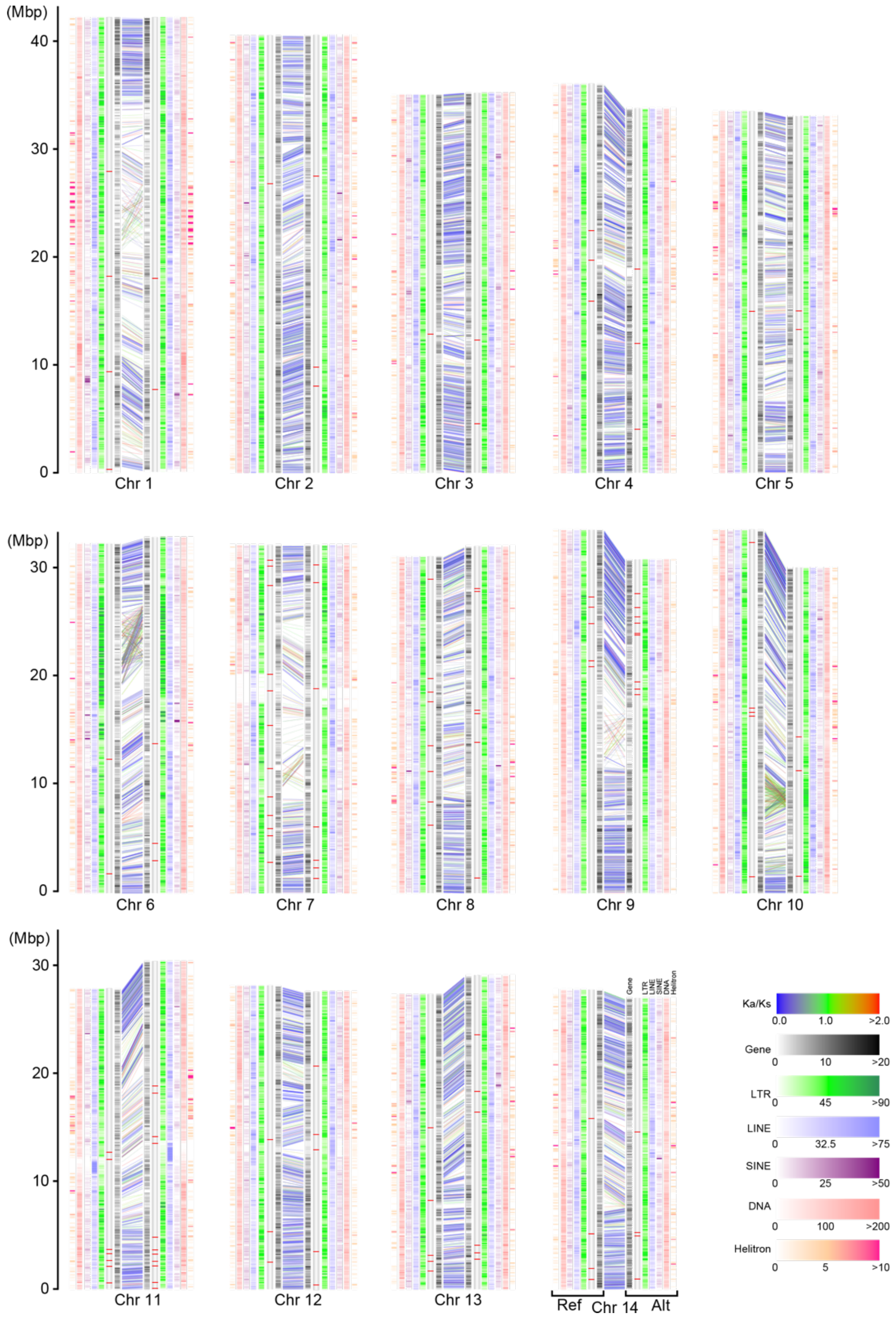

Supplementary Figure S7. Collinearity between the 14 chromosomal scaffold pairs of Ref and Alt haplotypes of *A. tenuis* characterized by syntenic gene arrangement, gene density, GC%, and density of transposable elements including LTR, LINE, SINE, DNA, and Helitron transposons. See also Figure 2.

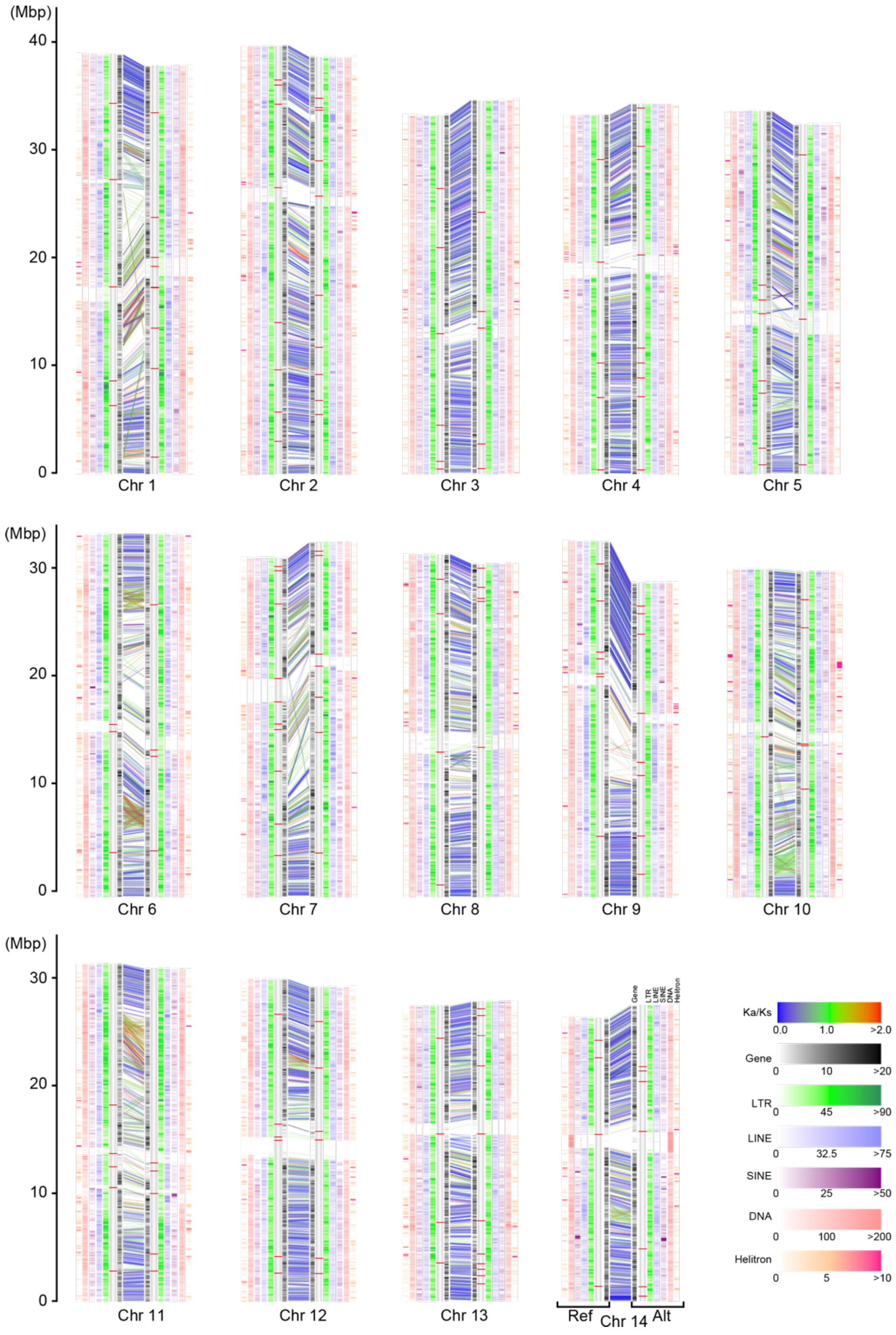

Supplementary Figure S8. Collinearity between the 14 chromosomal scaffold pairs of Ref and Alt haplotypes of *A. digitifera* characterized by syntenic gene arrangement, gene density, GC%, and density of transposable elements including LTR, LINE, SINE, DNA, and Helitron transposons. See also Figure 2.

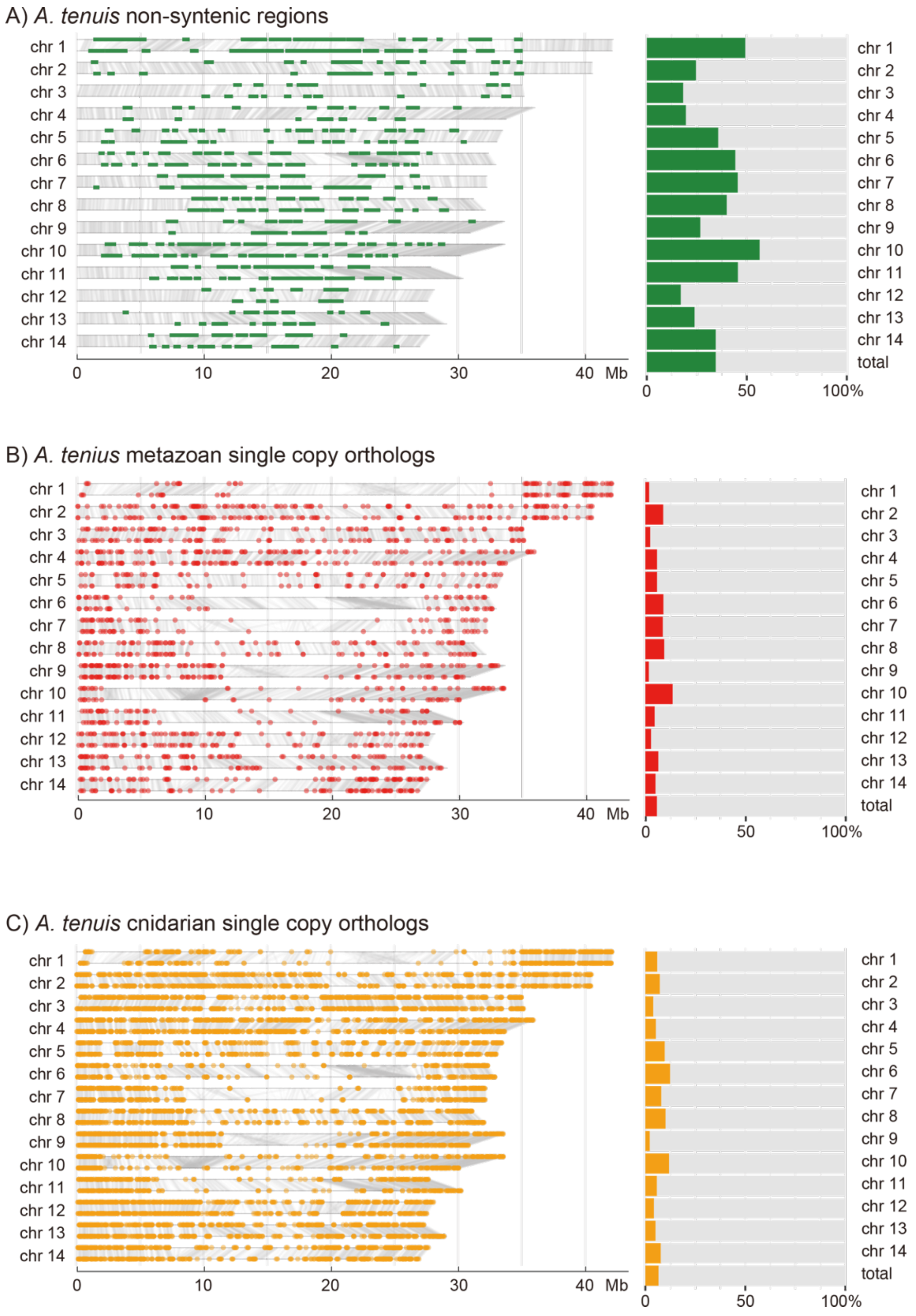

Supplementary Figure S9. Unequal distribution of nSRs and single-copy orthologs in *A. tenuis* genome. (A) Distribution of nSRs. The right bar plot indicates the percentage of nSRs in each chromosome. (B) Distribution of metazoan single-copy orthologs. (C) Distribution of cnidarian single-copy orthologs. The right bar plots of B and C indicate the percentage of SCOs in nSRs.

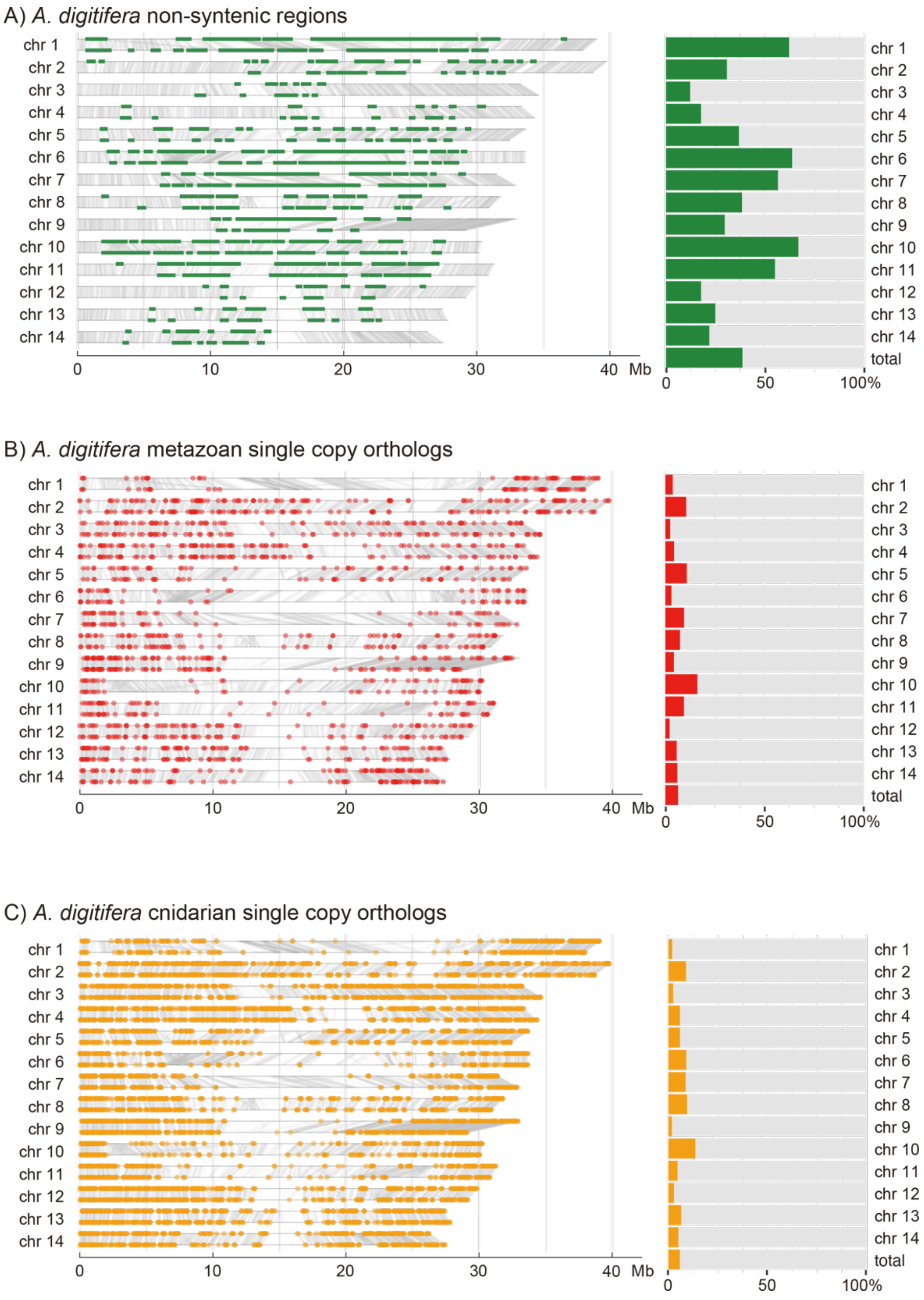

Supplementary Figure S10. Unequal distribution of nSRs and single-copy orthologs in *A. digitifera* genome. (A) Distribution of nSRs. The right bar plot indicates the percentage of nSRs in each chromosome. (B) Distribution of metazoan single-copy orthologs. (C) Distribution of cnidarian single-copy orthologs. The right bar plots of B and C indicate the percentage of SCOs in nSRs.

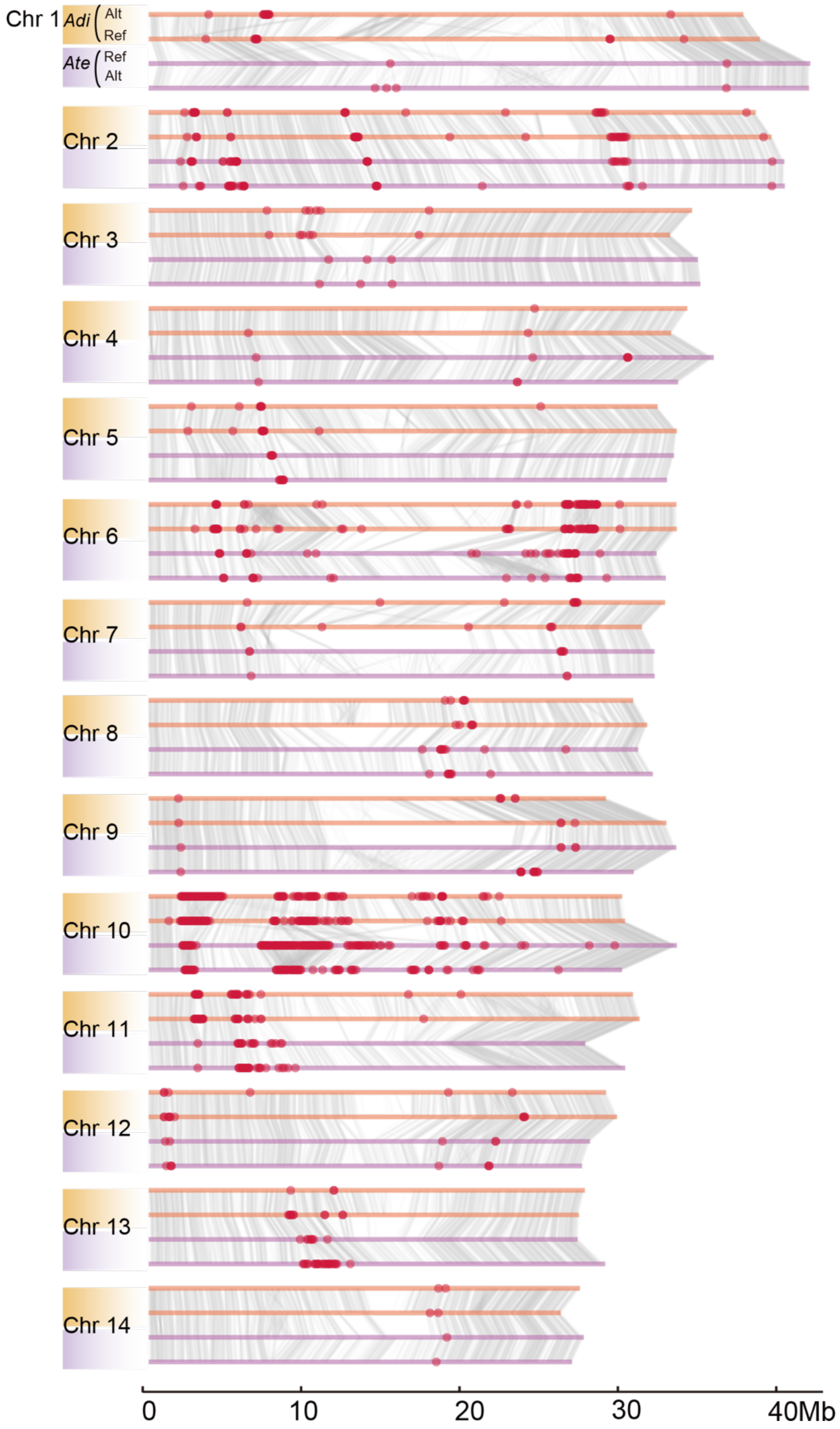

Supplementary Figure S11. Positions of TPR-CHAT DCP genes in *Acropora* genomes.

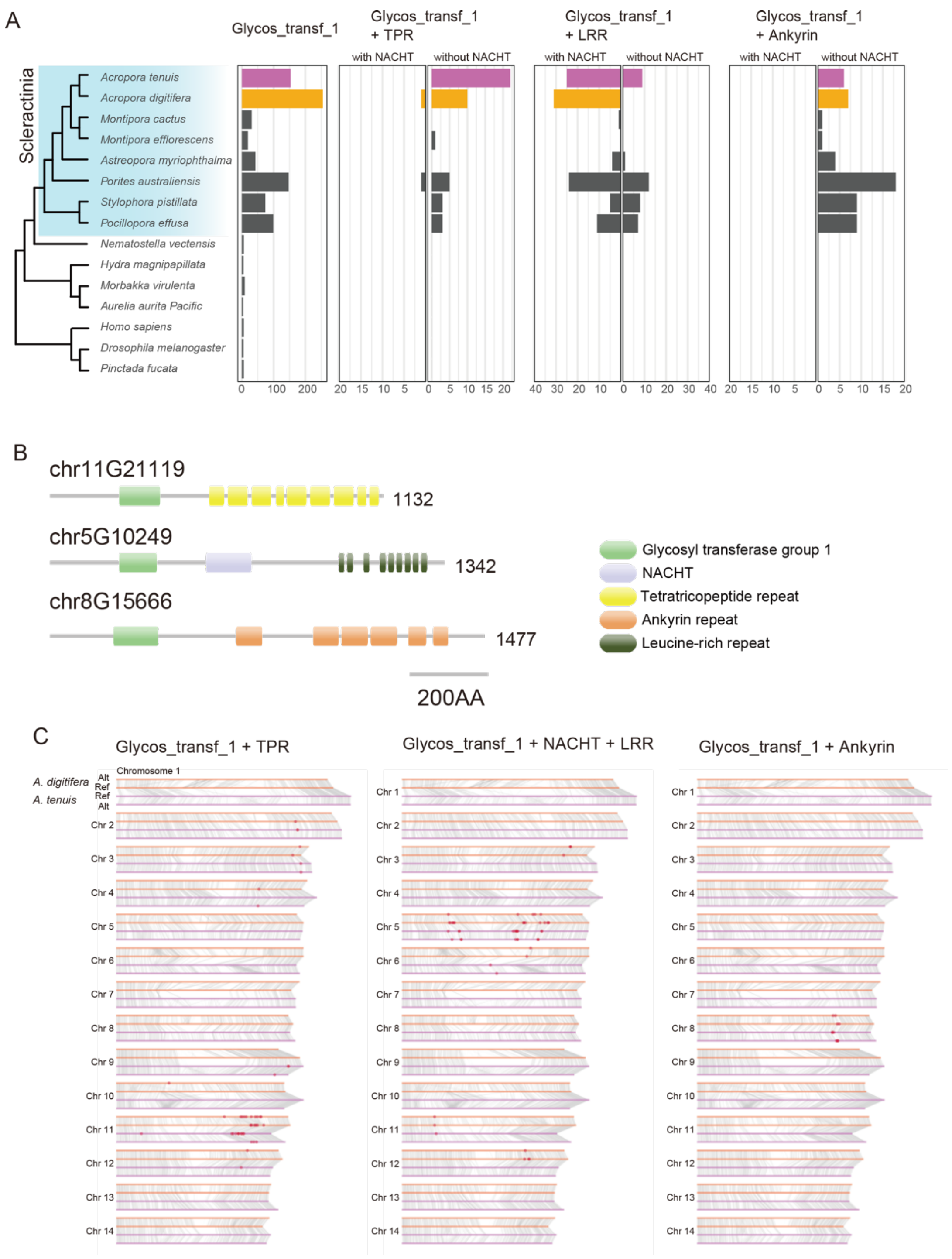

Supplementary Figure S12. Coral-specific gene family expansion of glycos_transf_1 DCPs. (A) Copy number of glycos_transf_1 DCP genes in animal genomes. (B) Functional domain architectures of glycos_transf_1 DCPs. (C) Positions of glycos_transf_1 DCP genes in *Acropora* genomes. Genes encoding different domain architectures are clustered in distinct nSRs.

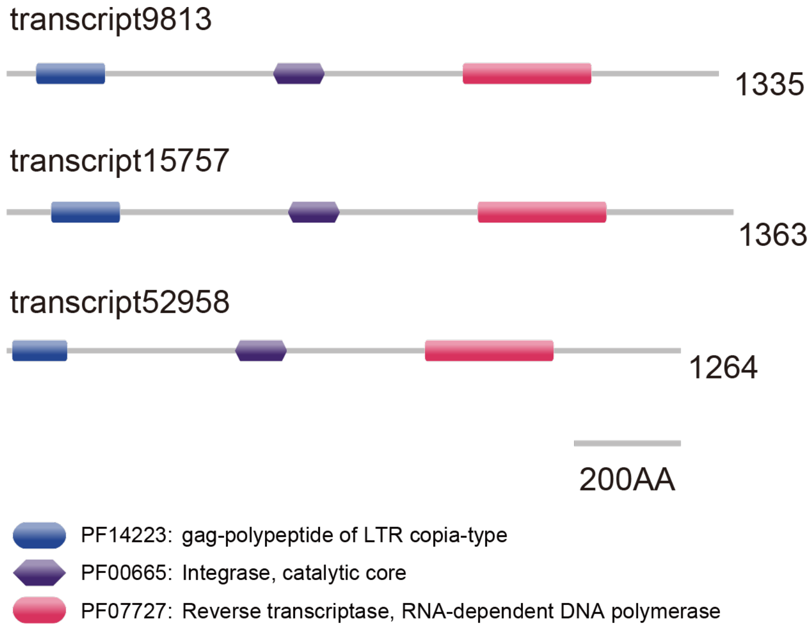

Supplementary Figure S13. Examples of transcript sequences of *Copia* transposons identified in Iso-seq reads.
